# Americans’ perceptions of privacy and surveillance in the COVID-19 pandemic

**DOI:** 10.1371/journal.pone.0242652

**Published:** 2020-12-23

**Authors:** Baobao Zhang, Sarah Kreps, Nina McMurry, R. Miles McCain

**Affiliations:** 1 Department of Government, Cornell University, Ithaca, NY, United States of America; 2 Institutions and Political Inequality Unit, WZB Berlin Social Science Center, Berlin, Germany; 3 Stanford University, Palo Alto, CA, United States of America; Universitat Luzern, SWITZERLAND

## Abstract

**Objective:**

To study the U.S. public’s attitudes toward surveillance measures aimed at curbing the spread of COVID-19, particularly smartphone applications (apps) that supplement traditional contact tracing.

**Method:**

We deployed a survey of approximately 2,000 American adults to measure support for nine COVID-19 surveillance measures. We assessed attitudes toward contact tracing apps by manipulating six different attributes of a hypothetical app through a conjoint analysis experiment.

**Results:**

A smaller percentage of respondents support the government encouraging everyone to download and use contact tracing apps (42%) compared with other surveillance measures such as enforcing temperature checks (62%), expanding traditional contact tracing (57%), carrying out centralized quarantine (49%), deploying electronic device monitoring (44%), or implementing immunity passes (44%). Despite partisan differences on a range of surveillance measures, support for the government encouraging digital contact tracing is indistinguishable between Democrats (47%) and Republicans (46%), although more Republicans oppose the policy (39%) compared to Democrats (27%). Of the app features we tested in our conjoint analysis experiment, only one had statistically significant effects on the self-reported likelihood of downloading the app: decentralized data architecture increased the likelihood by 5.4 percentage points.

**Conclusion:**

Support for public health surveillance policies to curb the spread of COVID-19 is relatively low in the U.S. Contact tracing apps that use decentralized data storage, compared with those that use centralized data storage, are more accepted by the public. While respondents’ support for expanding traditional contact tracing is greater than their support for the government encouraging the public to download and use contact tracing apps, there are smaller partisan differences in support for the latter policy.

## Introduction

As COVID-19 infections increased worldwide, governments adopted numerous public health surveillance measures, including those deploying new technologies. Digital contact tracing using smartphone apps has been endorsed by many governments, private firms, and universities as a potential tool to limit the spread of COVID-19. Traditional contact tracing involves identifying individuals who test positive for a disease, then identifying, locating, and isolating those who have come into contact with the infected individuals. While traditional contact tracing has been successful in countries like South Korea, China, Taiwan, and Singapore, efforts in the U.S. have lagged due to high caseloads, lack of testing, and distrust of contact tracers [1]. A Reuters investigation in August 2020 found that only about half of the local health departments with more than 1,000 cases reached close to all infected people [2]. In the U.S., the scale of the COVID-19 pandemic and the high rate of transmission have required a vast expansion of contact tracing to limit the spread of the disease [3, 4].

Given the shortage of human contact tracers and the time required to recruit and train sufficient numbers, some public health experts and technologists have recommended deploying contact tracing smartphone apps to supplement traditional contact tracing [5]. While states in the U.S. were slow to deploy contact tracing apps, many other countries did so early and with varying degrees of adoption. As of November 12, 2020, *MIT Technology Review*’s database of contact tracing smartphone apps had identified 47 countries that have introduced or are planning to introduce such an app [6]. Of these apps, 36 are built on Bluetooth proximity tracing technology. Bluetooth proximity tracing requires the public to download and use an app on their Bluetooth-enabled smartphones. Then, when an individual tests positive, she reports her status to the app either directly or through a medical authority. Using a record of anonymous key codes exchanged between phones, the app then alerts other users who have recently been in close proximity to the infected individual to ask them to quarantine.

Two versions of Bluetooth-based contact tracing apps have emerged. One approach involves centralized data storage such that when an infected person reports her status to the app, her anonymized I.D. *and* all the key codes that her phone has recently received are sent to a central database. A central server uses this database to perform contact matching and notify those who have recently come into contact with the infected person. Another approach—the one taken by the Google/Apple Exposure Notification System—employs a decentralized system, such that when an infected person reports her status to the app, only *her* anonymized I.D.s are added to the central list of infected individuals. Other users’ phones will download this list on a regular basis to check for previously seen I.D.s; contact matching is done locally on users’ phones rather than via a central server. The latter method preserves a higher degree of privacy. However, those developing or using centralized apps suggest that their approach allows governmental health authorities to manage the app better and gain more insights into the spread of the virus [7].

Understanding public perceptions of contact tracing apps is essential because broad uptake within a population is necessary for an app to be successful. Although the app can effectively reduce transmission at all levels of adoption, epidemiological modeling suggests that an app could stop the pandemic if the app were adopted by 60% of the population [8]. Indeed, the adoption of smartphone contact tracing may be an example of conditional cooperation, in which individuals are only willing to participate if they perceive others will participate as well [9].

We reviewed several studies that have examined public perceptions of contact tracing apps. These studies provide inconclusive evidence regarding Americans’ willingness to use contact tracing apps. Some surveys find that a large majority of Americans are willing to download and use contact tracing apps, including those with “opt-out” automatic installation [10–13]. Nevertheless, other surveys find that most Americans are unwilling to use contact tracing apps [14] or think that they will be ineffective at limiting the spread of COVID-19 [15]. The differences in the public’s acceptance across surveys may be attributable to the different features of the surveys’ hypothetical contact tracing apps [13]. In particular, given the frequent concerns individuals cite about privacy [11], the reluctance to download contact tracing apps may hinge on the privacy-preserving features of these apps [10, 13].

We surveyed approximately 2,000 U.S. respondents to understand their attitudes toward COVID-19 related surveillance and how they respond to different features of a hypothetical contact tracing app. Our research provides experimental evidence of how app features affect public support and potential uptake of the technology, using a design that allows direct comparisons in the importance of various design and policy choices. Our study speaks directly to the central policy debates around digital contact tracing, including whether public health authorities should adopt a decentralized or centralized data architecture. In addition, we put digital contact tracing in perspective by examining attitudes toward other types of public health measures such as temperature checks and traditional contact tracing and whether support for these measures is associated with demographic characteristics, political beliefs, and personal experience with COVID-19.

## Materials and methods

### Data

We conducted an approximately 15-minute survey between June 24 and June 25, 2020. (We conducted a pilot survey with 100 respondents on June 24 to troubleshoot the survey ahead of the main launch on June 25.) The analysis for this project was pre-registered using the Open Science Framework on June 25, 2020 before the main survey launch: https://osf.io/aemq6/. Lucid, an online survey firm, administered the survey. A total of 1,964 respondents completed the entire survey; 2,703 answered some part of the survey. The number of responses may vary between blocks of the survey, so we report the number of responses and the number of unique respondents in each figure and table.

**Fig 1:**
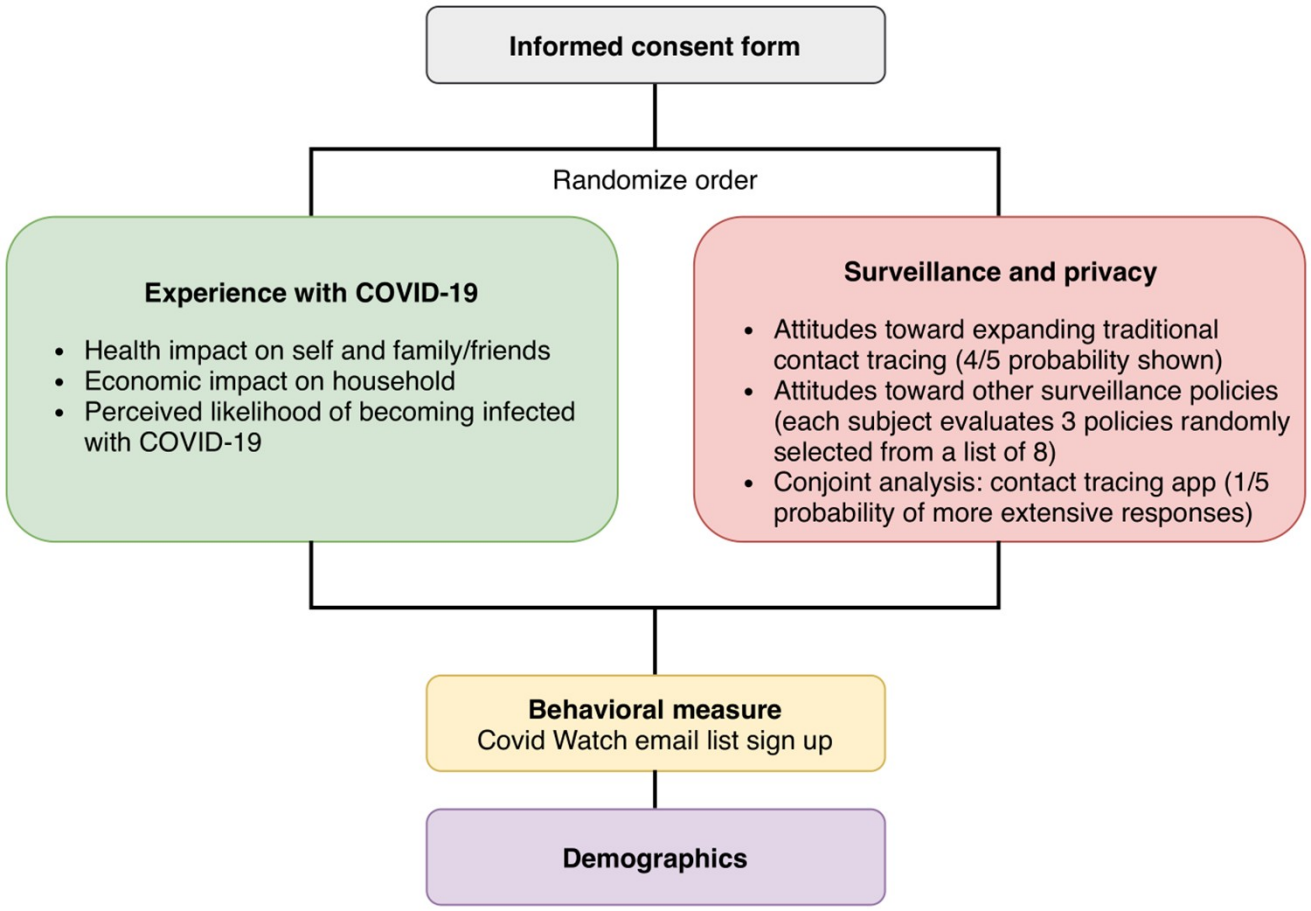
Survey flowchart. The figure illustrates the survey flow.

**Fig 2:**
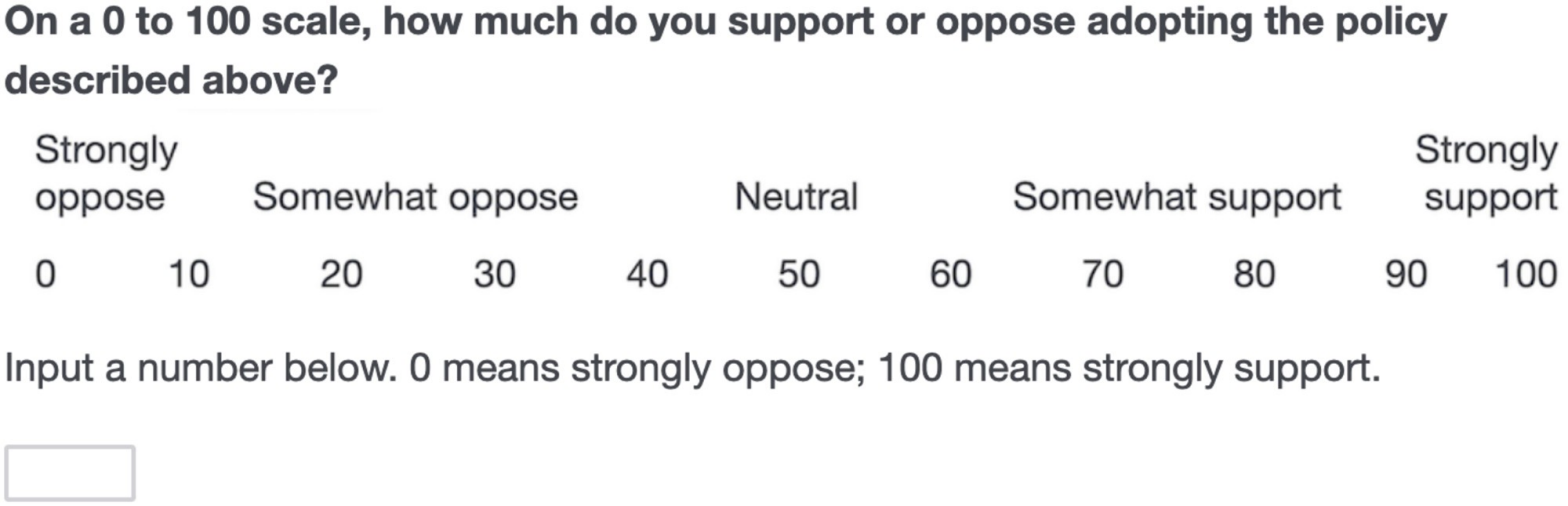
Survey response input for assessing policy support.

While Lucid does not provide probability samples of the U.S. adult population, its quota samples approximate the marginal distributions of key demographic characteristics. Recent validation exercises found that Lucid samples approximate nationally representative samples in terms of demographic characteristics and survey experiment effects [16]. We present our main descriptive results weighted to match marginal distributions of age, gender, region, race, income, and education in the U.S. adult population using data from the 2018 American Community Survey. Fig 2 in S1 File. shows the distribution of survey weights. Demographic summary statistics for our sample are included in Table 1 in S1 File. The flowchart (Fig 1) illustrates the structure of the survey. The text of the survey and the numerical coding of the multiple choice answers are found in S2. The replications files for this paper are available on Harvard Dataverse: https://doi.org/10.7910/DVN/5UEFP6.

### Priming experiment: Experience with COVID-19

After filling out the informed consent form, respondents who agreed to take the survey were randomized with equal probability into two different survey orders. We hypothesized that direct personal experience with COVID-19, such as having contracted the disease or knowing someone who has, would cause respondents to express higher levels of support for public health measures intended to limit the spread of the virus. To test this hypothesis experimentally, we randomly assigned respondents to a priming treatment. Those assigned to the priming treatment condition were presented with the section of questions related to their experience with COVID-19 before the section on surveillance and privacy. The “Experience with COVID-19” section asked about the health and economic impact of the virus on the respondent, as well as their perceived likelihood of becoming infected with the virus. Those in the control group saw the “Surveillance privacy” section of the survey before the “Experience with COVID-19” section.

Within the “Experience with COVID-19” section, we measured respondents’ level of confidence in several institutions or actors, including journalists, tech companies, and public health authorities, to act in the public’s best interest. Respondents were presented with three randomly-selected institutions or actors out of a total of seven and evaluated each using a 4-point scale (0 = no confidence at all; 3 = a great deal of confidence). Furthermore, we asked each respondent how much they trust COVID-19 related advice coming from Donald Trump, the CDC, and their state’s governor. Responses to this second trust question were measured using a 5-point scale (-2 = distrust a lot; 2 = trust a lot).

### Attitudes toward surveillance policies

The first block within the “Surveillance and privacy” section examined respondents’ attitudes toward expanding traditional contact tracing. Each respondent had a 4/5 probability of being shown this block (to avoid making the survey excessively long). Respondents not shown this block were instead presented with a block where they were asked to provide in-depth reflections of the hypothetical contact tracing app they read about in the conjoint analysis experiment.

Respondents were asked how much they supported or opposed the federal government or their state government expanding contact tracing. In this embedded mini-experiment on survey wording, each respondent had an equal probability of being asked about the federal government or their state government. The level of government (i.e., federal versus state) remained the same for each respondent when they were asked similar questions in subsequent blocks. Respondents used the 0 to 100 scale shown in Fig 2 to answer the question. We used a 0 to 100 scale instead of a 5-point Likert scale because we suspected that respondents would be averse to many of the surveillance policies described. A 5-point Likert scale would make it difficult for us to assess policy preference with nuance if most responses were clustered toward the lower end of the scale. Respondents input their responses into the textbox.

Using a matrix table format, we also asked respondents whether the policies would do the following:

- Improve the economy
- Make it safer for workers to return to work
- Make it safer for students to return to school
- Make it safer for me to visit friends and family
- Violate people’s privacy
- Violate people’s civil liberties
- Threaten U.S. democracy
- Make tech companies too powerful

The order of the potential consequences displayed was randomized. Respondents could select “yes,” “no,” or “I don’t know.”

The second block within this section measured respondents’ attitudes toward more invasive surveillance policies to limit the spread of COVID-19. Respondents were shown three of eight total possible policies, selected at random. They were told these policies have been adopted or considered for adoption in other countries. The eight policies that respondents considered include:

- Everyone would be required to have their temperatures taken (by no-touch forehead thermometers) before they are allowed to enter stores, workplaces, and other public spaces. Those who are found to have a fever would not be allowed to enter.
- Public health workers would be allowed to access people’s credit or debit card transactions for contact tracing if they have tested positive for COVID-19. This information would be shared with people who have been in the vicinity of a business where a transaction took place.
- Everyone who has tested positive for COVID-19 would be required to wear an electronic device to make sure that they are actually isolating. Those who do not comply could be ticketed and fined.
- Authorities would use CCTV cameras and drones to make sure that people are physically distancing in public spaces. Those who do not comply could be ticketed and fined.
- Authorities would encourage everyone to use an app on their smartphones that would record their recent in-person encounters. If someone tests positive for COVID-19, the apps would figure out who among their close contacts may have been infected in the days before and notify them to isolate.
- Authorities would set up thermal cameras (cameras that measure how much heat people give off) in public places to discourage those with a fever from going outside. Those who have a fever and are caught on thermal cameras could be ticketed and fined.
- Those who have tested positive for COVID-19 would be required to stay at a central isolation location (like a dorm or a field hospital), where medical staff would monitor their health. They would not be allowed to go home until they are cleared from isolation by the appropriate public health authorities.
- To use public transit or travel by train/plane, everyone would be required to show a government-issued certification on their cellphones confirming that they are not infectious.

The order in which the three policies were presented to each subject was randomized. After reading about each policy, respondents were asked how much they support or oppose the federal government or their state government adopting it using the same 0 to 100 scale in Fig 2. They were also presented with the same matrix table of potential consequences of the policy. Finally, for each policy respondents considered, they had a 1/3 probability of being asked to write their thoughts about the policy in a text box. For these open-ended responses, respondents were asked to consider the potential benefits and downsides of the policy.

### Conjoint analysis: Features of contact tracing apps

The final block in the “Surveillance and privacy” section of the survey included a conjoint experiment to determine how various features of contact tracing apps affect public perception. Conjoint analysis is a survey-based statistical tool often used in market research to study how consumers value different attributes of a product or service [17]. In a conjoint analysis study, respondents are presented with hypothetical products or services with randomized attributes. Recently, social scientists have applied this research method to study political or policy questions, such as how voters evaluate characteristics of electoral candidates or how citizens evaluate immigrants applying for citizenship [18].

In our study, we asked respondents to consider a contact tracing app that would notify them if they had recently come into close contact with someone who tested positive for COVID-19. We varied six attributes of the hypothetical app: the app developer, the app name, the data storage architecture, the conditions under which the app would expire, the minimum percentage of U.S. smartphone users who would need to use the app for it to be effective, and the technology used. These features were selected based on existing concerns around contact tracing apps. Table 1 shows the different features within each feature type.

**Table 1:** Conjoint analysis: Features of contact tracing app.

Feature type	Feature options
App developer	(1) Apple and Google; (2) the Centers for Disease Control and Prevention (CDC); (3) respondent’s state government; (4) researchers at leading universities
App name	(1) contact tracing; (2) exposure notification
Data storage	(1) decentralized: data stored on users’ phones, data better protected, health authorities have less ability to manage and analyze data; (2) centralized: data stored on a central server, data less protected, health authorities have more ability to manage and analyze data
Expiration	(1) after the CDC declares the pandemic over; (2) when a successful vaccine has been discovered; (3) no expiration date information given
Percentage of smartphone users needed to limit the spread of infections	(1) at least 60%; (2) at least 80%*
Technology used	(1) GPS that tracks users’ location; (2) Bluetooth that does not track users’ location; (3) Bluetooth that does not track users’ location + visual explainer of decentralized Bluetooth tracing technology

* In 2019, 79.1% of the U.S. population are estimated to own a smartphone [19]. Therefore, 60% of U.S. smartphone users are 48% of the U.S. population; 80% of U.S. smartphone users are 63% of the U.S. population. The higher percentage we tested in our conjoint analysis is close to the 60% number in the popularly-cited epidemiological model that predicts if 60% of the whole population downloads and uses the app, the pandemic could be stopped [8]. The lower percentage we tested is within the 20% to 56% range the model predicts would slow the spread of infections [20].

Given the large amount of information presented to respondents, our conjoint analysis differed from the usual format of a table where the rows are hypothetical attributes and the columns are candidates. Instead, we described the app in a short news article. To avoid overwhelming the respondents, each respondent read about only one app. Furthermore, each respondent had a 1/3 probability of seeing an illustration from a BBC news article explaining how decentralized Bluetooth proximity tracing, as implemented by the Google/Apple Exposure Notification System, works. (Respondents were required to spend at least 30 seconds reading the article before they could advance to the next page in the survey.) Fig 1 in S1 File shows an example of an article that respondents read. After reading about the app, respondents were asked a series of questions about the app. When answering these questions, respondents were able to refer back to the article, which was embedded within an accordion (i.e., collapsible content) on all survey pages about the app.

First, we assessed the respondents’ comprehension of the article by asking them to select true statements about the app. Twenty-percent of respondents (those who did not answer the traditional contact tracing question) were also asked to provide further reflections about the app, including open-ended responses.

Second, we assessed the respondents’ attitudes toward the contact tracing app. We asked those who reported having a smartphone how likely they would be to download and use the app, as well as how likely they would be to report their status to the app if they tested positive for COVID-19. Next, we requested all respondents to estimate the percentage of smartphone users in their town or city who would use the app. Respondents answered these three questions on a 0 to 100 point scale, referring to probability in the first two questions and estimated percentage in the third. Next, respondents were asked about 1) potential consequences if enough people used the app using the matrix table from the surveillance policy questions and 2) how confident they were that their data would be protected when using the app.

Finally, respondents were given a series of five statements (in randomized order) and asked how much they agreed or disagreed with these statements using a 5-point Likert scale. The statements included:

- {The federal government/your state government} should require everyone who has a smartphone to use this app.
- Apple and Google should automatically install this app on users’ iPhones or Android phones as part of a software update.
- Employers should require their employees with smartphones to use this app.
- Places of religious worship, like churches, synagogues, and mosques, should require everyone who has a smartphone to use this app if they want to worship there.
- App users should have a choice in sharing their test outcomes with the app if they tested positive for COVID-19.

### Demographics

At the end of the survey, respondents were asked to provide demographic information (e.g., age, gender, race, level of education, household income, location, party identification, and political ideology). This is not the ideal placement for the series of demographic questions because the previous questions might have affected respondents’ answers to the demographic questions. Nevertheless, we decided to place the main questions at the beginning of the survey instead of the demographic questions to prevent respondents from becoming fatigued before answering the main questions. Furthermore, Lucid provided us with the respondents’ basic demographic information from their own records, making it possible to verify respondents’ answers to some demographic questions.

## Results

### Observational study results: Attitudes toward public health surveillance policies

**Fig 3:**
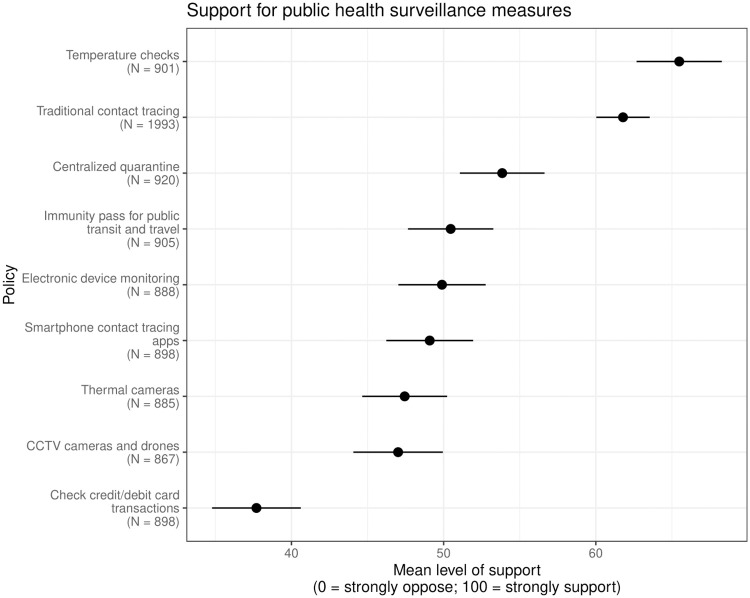
Mean levels of support for government adoption of surveillance policies among all respondents with 95% confidence intervals. Each respondent had a 4/5 probability of being asked about the expansion of traditional contact tracing. All respondents were asked about three of the other eight policies, randomly selected. Respondents were asked to indicate their level of support for each policy on a scale from 0 to 100, where 0 indicated strong opposition and 100 indicated strong support. Responses are weighted to the U.S. adult population. Point estimates and robust standard errors are listed in Table 2 in S1 File.

Fig 3 shows mean levels of support for the nine surveillance policies mentioned in the survey. Requiring temperature checks in public places garnered the highest mean level of support: 65 out of a possible score of 100. Respondents were least supportive of governments tracking credit or debit card transactions (with a mean score of 38 out of 100).

Smartphone contact tracing apps ranked sixth by mean level of support, with an average score of 49 out of 100 (well below temperature checks and traditional contact tracing). Using a dichotomous version of the variable, where a score of 60 out of 100 or above is coded as “somewhat support” or “strongly support” (consistent with the labels on the scale), we estimated that 42% of the population supported the adoption of these apps. Numerical estimates for each policy, including the continuous and binary versions of the outcome variable, are presented in Table 2 in S1 File.

#### Breakdown by demographic subgroups

We also examined differential support for surveillance policies based on respondents’ demographic characteristics. Fig 3 in S1 File shows demographic predictors of support for surveillance policies overall. Compared with male ones, female respondents were less supportive of the policies (two-sided *p* < 0.03). Democrats were more supportive of these measures compared with Independents (two-sided *p* < 0.001) and Republicans (two-sided *p* < 0.001). Fig 5 in S1 File shows the support for each policy by party identification. Democrats, compared with Republicans, were significantly more supportive of temperature checks, traditional contact tracing, and using CCTV cameras and drones to enforce social distancing. Partisan differences were less salient when it comes to the government encouraging everyone to use contact tracing apps. We found the 47% of Democrats, compared with 46% of Republicans, support or strongly support this policy. Significant partisan differences emerged in terms of opposition to digital contact tracing, however. Whereas 39% of Republicans oppose this policy, only 27% of Democrats opposed this policy.

Those aged 30-44, compared with those aged 18-29, were more supportive of the policies (two-sided *p* = 0.01). Those who make $100,000 or more annually, compared with those who make less, were more supportive of these policies (two-sided *p* < 0.001). Those with a bachelor’s degree or higher, compared with those with less than a high school degree, were more supportive (two-sided *p* < 0.001).

As shown in Table 3 in S1 File, respondents who had health conditions that would increase their risk for COVID-19 complications, who reported knowing someone who has tested positive for the virus (including themselves), or who had higher scores on indices capturing health and economic impact of the virus demonstrated significantly higher support for surveillance policies, controlling for demographic characteristics. Per the pre-analysis plan, we constructed two indices of personal COVID-19 impact. The health impact index aggregates whether the respondent or someone the respondent knows has tested positive for the virus, and whether the respondent knows someone who has died due to complications from COVID-19. The economic impact index includes whether the respondent or someone in the respondent’s household has lost their job or had work hours reduced as a result of the pandemic, whether the respondent thinks it is likely or very likely they or someone in their household will face loss or reduction in employment, and whether the respondent thinks their personal financial situation is getting worse.

#### Association between trust in institutions/actors and support for the policies

Fig 16 in S1 File. presents the Pearson correlation between trust in institutions/actors and support for each of the surveillance policies. These bivariate correlations show that trust in governmental, scientific, and civil society institutions/actors was positively correlated with support for each public health surveillance measure. There is one notable exception: trust in COVID-19 related advice coming from Donald Trump has very weak positive correlations (and even some negative correlations) with support for the policies. We investigated further using multiple regression, reported in Table 11 in S1 File. Even after controlling for demographic variables and the type of surveillance policy, general trust in institutions/actors and trust in COVID-19 related advice from the CDC and one’s state governor was associated with greater support for the policies.

#### Mini-experiment: Level of government enacting the policies

**Fig 4:**
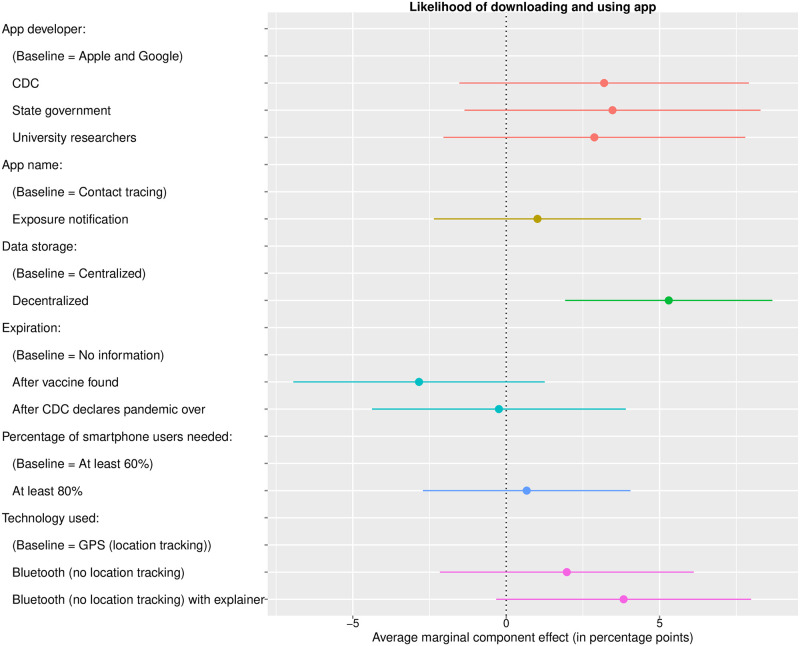
Average marginal component effects of contact tracing app attributes on reported likelihood of downloading and using the app with 95% confidence intervals. Respondents were shown a description of a hypothetical contact tracing app that varied along six attributes. The value shown for each attribute was selected randomly. Respondents were then asked how likely they would be to download and use the app described. This outcome is measured using a continuous scale from 0 to 100. The question was asked of all respondents who reported owning a smartphone (*N* = 1, 883). Coefficients depicted in the plot indicate the average marginal component effect of being shown a particular value of an attribute, compared to a baseline category, on the reported likelihood of downloading the app.

Fig 4 in S1 File shows support for surveillance policies disaggregated by the level of government that respondents were told would implement them (state or federal). The level of government for each policy was randomly assigned to each respondent. We did not detect any significant differences in support depending on the level of government. Table 4 in S1 File shows estimates of heterogeneous effects by party-identity and governor co-partisanship. We did not observe statistically significant interaction effects based on co-partisanship or party.

### Conjoint analysis: Features of contact tracing apps

Results from the conjoint experiment examining the effects of hypothetical app features on respondents’ inclination to download the app are presented in Fig 4. The main outcome captured the self-reported likelihood of downloading the described contact tracing app on a scale from 0 to 100. This question was asked only of respondents who reported owning a smartphone (*N* = 1, 883, or 96% of those who completed the entire survey). Additional outcomes include the respondents’ likelihood of reporting a positive test result to the app (limited to smartphone users), confidence that their data will be protected (asked of all respondents), and estimated percentage of people in the respondent’s city or town they believe would download the app (asked of all respondents). Results for these outcomes appear in Figs 10 to 12 in S1 File, respectively.

Averaging across all profiles, the mean likelihood of downloading a hypothetical app was 47. Using a dichotomous version of the variable (coding a score of 60 or above as “likely to download”), we estimated that 42% of respondents indicated that they would download and use such an app. These estimates are generally consistent with our estimates of support for the government’s encouraging everyone to use smartphone contact tracing in the previous block of the survey.

Of the six randomly-varying attributes, only one had statistically significant effects on the self-reported likelihood of downloading the app: the data architecture. The likelihood of downloading and using an app with decentralized data architecture, compared with the likelihood for an app with centralized data architecture, is 5.4 percentage points higher on average. Differences in the remaining attributes—the identity of the app developer, the app name, the condition for app expiration, the percentage of the population needed for the app to be effective, and the type of technology used—had no statistically significant effect at the 5%-level on the likelihood. Figs 8 and 9 in S1 File show average marginal component effects and marginal means using the dichotomous outcome (60% or greater likelihood). The decentral-ized data storage feature also increased respondents’ willingness to report their status to the app if they test positive (see Fig 10 in S1 File) and their confidence in the security of their data (see Fig 11 in S1 File).

#### Manipulation check

We found evidence of considerable misunderstanding about contact tracing apps even after informing respondents about them. As part of the conjoint analysis, we performed a manipulation check to see if respondents could correctly identify features of the apps they had just read about. Respondents were given a list of five statements and asked to select all the true statements about the app. Respondents had access to the description of the app (available as collapsible content) while answering this question. On average, they gave the correct answer to 3.2 out of the five statements. (See Table 14 in S1 File for the percentage of correct responses to each statement). For example, although all of the app descriptions mentioned that the app would not identify individuals by name, 31% of respondents still marked the statement “the app will send you the names of infected people you have been in close contact with” as true.

News articles about contact tracing apps have sometimes used visual explainers to help readers understand how Bluetooth proximity tracing apps work. In the conjoint analysis, we tested a treatment that included a visual explainer from the BBC. However, as Fig 13 in S1 File shows, including the visual explainer did not affect respondents’ accuracy in the manipulation check. These results suggest that there is significant work to be done in public health communication to ensure the public understands how contact tracing apps work.

We performed additional analysis to understand the determinants of wrong answers in the manipulation check. As Table 12 in S1 File shows, expressing greater support for public health surveillance policies, identifying as not white, Black, or Latinx, and making greater than $99,000 in household income annually were all associated with more incorrect answers. Identi-fying as female, being in the 45-59 age group, and having some college education or a bache-lor’s degree or higher were all associated with fewer incorrect answers. Giving more incorrect answers is not associated with greater opposition to downloading and using the app or reporting positive results to the app, as seen in Table 13 in S1 File. Interestingly, respondents with more wrong answers expressed greater agreement with the statement that the government should require smartphone users to download and use the app, even controlling for demographic variables.

#### Attitudes toward contact tracing app policies

Respondents were presented with five hypothetical contact tracing app policies and asked how much they agreed or disagreed with them. The aggregate results and the marginal means by app feature are presented in Table 6 through 10 in S1 File. Overall, 36% agreed that the government should require everyone with a smartphone to use the app; 39% agreed that employers should require employees with smartphones to use the app; 34% agreed that Apple and Google should automatically install the app on users’ smartphones as part of a software update; and 37% agreed that places of religious worship should require individuals to use the app if they wish to worship there. A majority of respondents (58%) agreed that app users should have a choice in sharing their test outcomes with the app if they test positive for COVID-19.

The features of the contact tracing apps did little to change respondents’ attitudes toward these policies. The decentralized data storage feature slightly increased respondents’ support for the government, employers, and places of religious worship to require smartphone users to use the app, but only for places of religious worship was the effect statistically significant at the 5%-level.

#### COVID-19 experience priming experiment

**Fig 5:**
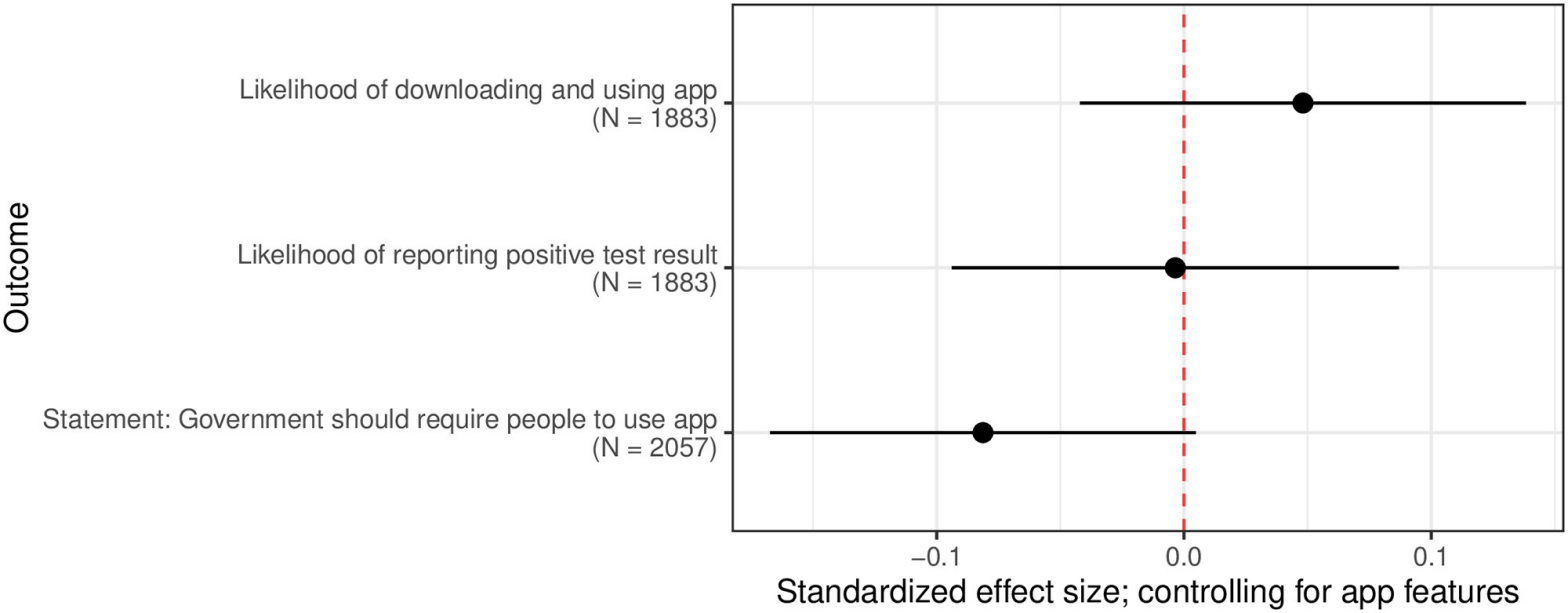
Estimated effects of COVID-19 experience question section priming on support for smartphone apps, with 95% confidence intervals. A randomly selected half of respondents were asked a section of questions regarding their own experiences with COVID-19 prior to questions regarding their support for a hypothetical contact tracing app (the “prime” condition), while the other half were asked these questions afterward (the “control” condition). The quantities depicted show the estimated effects of being assigned to the prime condition, compared to the control condition, on the various measure of support for the contact tracing app. Coefficients come from a saturated linear regression controlling for hypothetical app features.

Fig 5 shows the estimated effects of the priming treatment on three measures of support for a hypothetical contact tracing app: likelihood of downloading the app, likelihood of reporting a positive test result to the app, and agreement that the government should require everyone with a smartphone to use the app. We did not find evidence supporting the hypothesis that priming respondents with questions regarding their personal experience with the COVID-19 pandemic increases support for smartphone contact tracing. Given the extent of harm caused by the virus, it is likely that respondents’ own experiences were immediately cognitively accessible upon being asked questions related to the pandemic, even when they were not asked about these experiences directly. As discussed above, we found in our observational analysis significantly higher levels of support for surveillance measures, including smartphone contact tracing, for individuals who have been personally affected by the pandemic.

## Discussion

Our research on public attitudes toward COVID-19 public health surveillance in general and digital contact tracing in particular yields five main findings. First, 42% of respondents supported the government encouraging everyone to use contact tracing apps. Support for this policy was lower than support for expanding traditional contact tracing. Second, apps using decentralized data storage, compared with those using centralized data storage, were more likely to be accepted. In our conjoint analysis experiment, we found that 44% of respondents would download a hypothetical app that stores data locally on users’ phones, compared to 39% when the app was described as storing data on a centralized server. Third, individuals with pre-existing health conditions or who had been personally affected by the virus were more likely to support contact-tracing apps and expanded surveillance in general. This finding is consistent with the Health Belief Model [21], which proposes that, among other reasons, individuals are more likely to follow health recommendations if they feel they are both susceptible to an illness and that the illness is potentially severe. It suggests that support may grow as the virus spreads and more Americans personally experience its effects. Fourth, we found differences in support for contact tracing apps between Democrats and Republicans were relatively small, indicating that certain politically-aligned resistance to quarantine and mask mandates [22] may not extend to smartphone contact tracing yet. However, Independents were more skeptical of apps and of public health surveillance measures generally. Finally, we found evidence that the public holds misinformed beliefs about contact tracing apps even after reading about how the apps work. Nevertheless, these misinformed beliefs as a whole were not associated with opposition to downloading and using the apps.

This research is the first to our knowledge that evaluates public attitudes about digital contact tracing alongside other public health surveillance measures. While it offers insights into preferences about key public health measures, the study does have limitations. One is that we recruited respondents through Lucid, whose quota samples approximate nationally representative samples in terms of demographic characteristics and survey experiment effects [16]. Additional studies should consider using probability-based sampling to replicate the results. Furthermore, our survey captures the U.S. public’s attitudes toward public health surveillance measures at only one point in time. Research has shown that Americans’ COVID-19 related attitudes have shifted over time; for instance, the percentage of those who reported wearing face masks has increased [23]. We plan to re-survey the respondents of this study to examine how their attitudes have changed over time. Finally, we note that self-reported willingness to download and use the apps may be higher than actual behavior due to experimenter demand effects. The actual usage of contact tracing apps in the U.S. may be lower than that reported in our paper.

Despite these limitations, our findings may help inform public health authorities to increase participation in digital contact tracing apps. Given the considerable public reluctance about the technology, governments considering deploying digital contact tracing apps should attempt to alleviate these concerns by adopting privacy-preserving technology. In addition, any deployment must be accompanied by a public education campaign to emphasize that 1) Bluetooth proximity tracing does not record location data, 2) the individual identities of users are never collected, and 3) decentralization means that contact data is not transmitted to a central server. Further, our finding that support for these apps is not yet very polarized along partisan lines suggests that bipartisan elite cues could be useful in boosting public support for download and use.

Privacy-preserving technology and public education are both necessary for a digital contact tracing app to be effective, but are not themselves sufficient. Many of the populations that are most vulnerable to COVID-19, such as those who are elderly, low-income, or homeless, also have lower cell phone ownership and, therefore, will not benefit from smartphone contact tracing apps [24]. Thus, public health officials will need to redouble efforts to ensure these populations’ safety, including expanding testing and traditional contact tracing. In addition, Fig 6 in S1 File suggests that Black Americans, a group already disproportionately affected by the pandemic, are less supportive of expanding traditional contact tracing compared with white Americans. Efforts to introduce digital contact tracing must address marginalized communities’ concerns, such as the fear that law enforcement could access user contact data. Furthermore, legislators may alleviate public concerns about data misuse by enacting legislation to protect user data, such as that passed by Australia’s House of Representatives [25], and by requiring third-party audits of the apps [26].

While digital contact tracing apps are a promising tool to limit the spread of COVID-19, we found that public perceptions of digital contact tracing are a barrier to widespread adoption. Therefore, governments looking to deploy digital contact tracing must invest not only in the app itself but also in a significant public education campaign to encourage adoption. Finally, contact tracing apps must not be treated as a standalone solution to the pandemic; instead, they must be part of a greater public health strategy that is mindful of all populations’ needs and concerns.

## Supporting information

S1 File Supporting information file. The supporting information file provides additional details about our experiments, including demographic details, disaggregated responses, and survey information.
(PDF)

S2 File Survey instrument. The survey instrument file provides detailed information about the questions, branching conditions, and consent options presented to respondents.
(PDF)
