# Supplementary material for: Americans’ perceptions of privacy and surveillance in the COVID-19 pandemic
Source: PLoS One. 2020 Dec 23;15(12):e0242652. doi: 10.1371/journal.pone.0242652 (PMC7757814; doi:10.1371/journal.pone.0242652)
Supplement: S1 File — The supporting information file provides additional details about our experiments, including demographic details, disaggregated responses, and survey information. (PDF) [file pone.0242652.s001.pdf]

Americans' perceptions of privacy and surveillance in the COVID-19 Pandemic

Baobao Zhang, Sarah Kreps, Nina McMurry, and R. Miles McCain

November 19, 2020

Table 1: Sample demographics.

Variable	Value	Proportion	Count
Age	18-29	0.293	792
Age	30-44	0.266	720
Age	45-59	0.215	581
Age	60+	0.226	610
Gender	Female	0.546	1476
Gender	Male	0.454	1227
Income	$100K+	0.158	428
Income	$50K-90K	0.211	569
Income	<$50K	0.564	1525
Income	Income NA	0.067	181
Education	BA or higher	0.393	1063
Education	Did not graduate HS	0.050	134
Education	HS graduate	0.272	735
Education	Some college	0.267	723
Education	Education NA	0.018	48
Race	2+ Races, Non-Hispanic	0.012	32
Race	Black, Non-Hispanic	0.138	374
Race	Hispanic	0.134	363
Race	Other, Non-Hispanic	0.066	179
Race	White, Non-Hispanic	0.623	1683
Race	Race NA	0.027	72
Region	Midwest	0.188	507
Region	Northeast	0.211	570
Region	South	0.380	1026
Region	West	0.222	600

Fig. 1: Sample conjoint analysis survey page as it appeared to respondents.

The Centers for Disease Control and Prevention (CDC) is building a contact tracing app for smartphones. It will be used to identify and alert people who have come into contact with those infected with COVID-19. The app will recommend those who came into contact with infected persons to self-isolate for two weeks.

The app uses Bluetooth data that does not track users' locations. The app will not reveal the identity of infected persons. Here is how the app works:

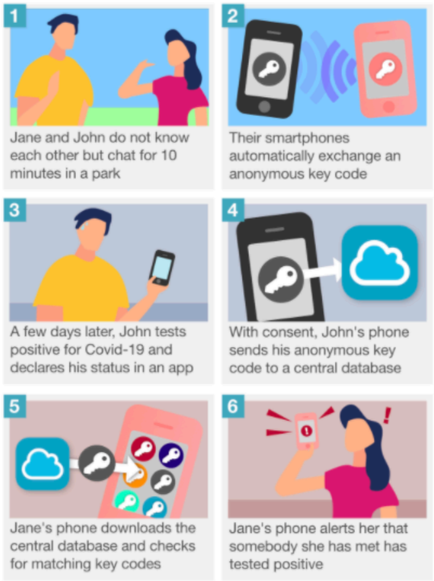

All user data will be stored on a central server controlled by the app developer.

The centralized data storage would give public health authorities greater ability to manage and analyze user data. However, this setup makes users' data less protected against intrusions by hackers or the government.

By storing data on a central server, users must unlock their phones and have the app open for the app to work. While the app is running, users cannot make/take calls or use other apps.

Public health experts say that at least 80% of US smartphone users need to use this app for it to be effective at limiting the spread of COVID-19.

The app would expire after the Centers for Disease Control and Prevention (CDC) declares the COVID-19 Pandemic is over.

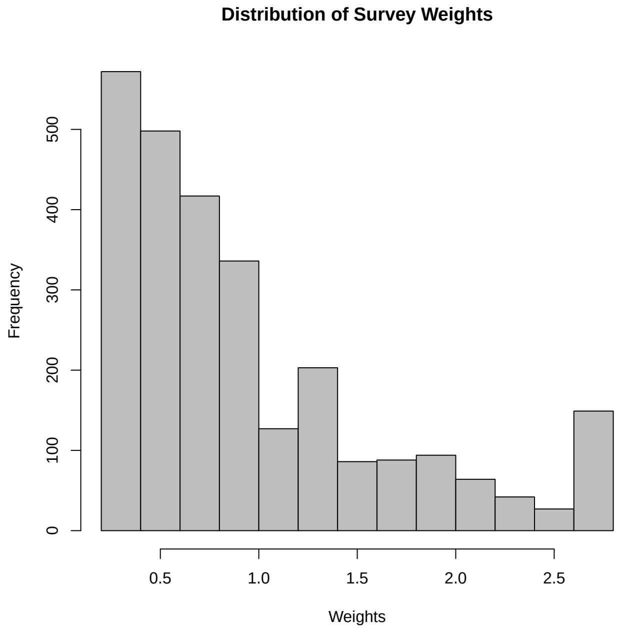

Fig. 2: Distribution of survey weights. Target proportions for age, gender, region, race, and income come from the 2018 American Community Survey. Weights were calculated using iterative proportional fitting and trimmed at the 5th and 95th percentiles. Missing demographic information was imputed for the purpose of generating sample weights using multivariate imputation by chained equations (MICE).

Table 2: Support for public health surveillance measures: summary of responses.

Outcome	Mean level of support	SE	Percent support/strongly support	N	Proportion missing
Traditional contact tracing	61.78	0.90	56.87	1993	0.01
Temperature checks	65.48	1.43	61.88	901	0.01
Check credit/debit card transactions	37.70	1.49	31.22	898	0.02
Electronic device monitoring	49.89	1.46	43.61	888	0.02
CCTV cameras and drones	47.01	1.50	40.07	867	0.01
Smartphone contact tracing apps	49.08	1.46	42.07	898	0.02
Thermal cameras	47.44	1.42	39.54	885	0.01
Centralized quarantine	53.85	1.42	48.79	920	0.02
Immunity pass for public transit and travel	50.46	1.43	43.45	905	0.01

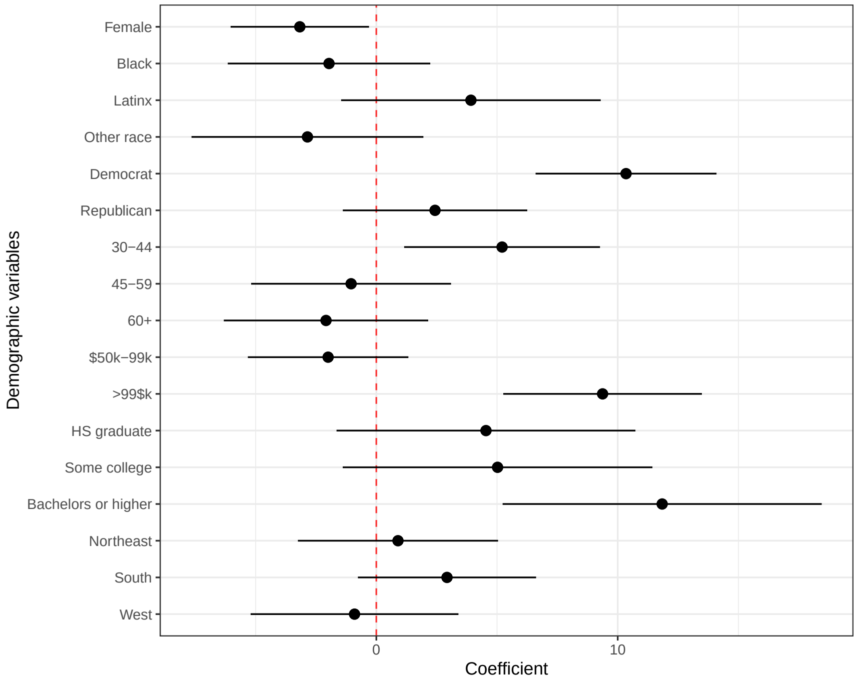

Fig. 3: Demographic predictors of support for surveillance policies, measured on a scale from 0 to 100. Each respondent was asked to rate their support for four policies: traditional contact tracing and three randomly selected among the remaining options. Plot shows coefficients from a single OLS regression of support for any surveillance policy on all categorical demographic characteristics listed, with 95% confidence intervals. Standard errors are clustered at the level of the respondent ($N = 2,511$). Baseline categories are as follows: male (gender), white (race), Independent/other (party), 18-29 (age), less than $50k per year (income), did not graduate high school (education), Midwest (region).

Table 3: COVID-19 experience and support for surveillance.

	Dependent variable:			
	Policy support			
	(1)	(2)	(3)	(4)
Comorbidity	7.642***			
	(1.579)			
Know COVID positive		4.542**		
		(1.479)		
Health impact index			3.172***	
			(0.643)	
Economic impact index				1.709**
				(0.657)
Demographic controls	Y	Y	Y	Y
Respondents	2,509	2,509	2,509	2,509
Observations	9,038	9,038	9,038	9,038
R^2	0.091	0.085	0.090	0.084
Adjusted R^2	0.089	0.083	0.088	0.082

Cluster robust standard errors at respondent level — *p<0.05; **p<0.01; ***p<0.001

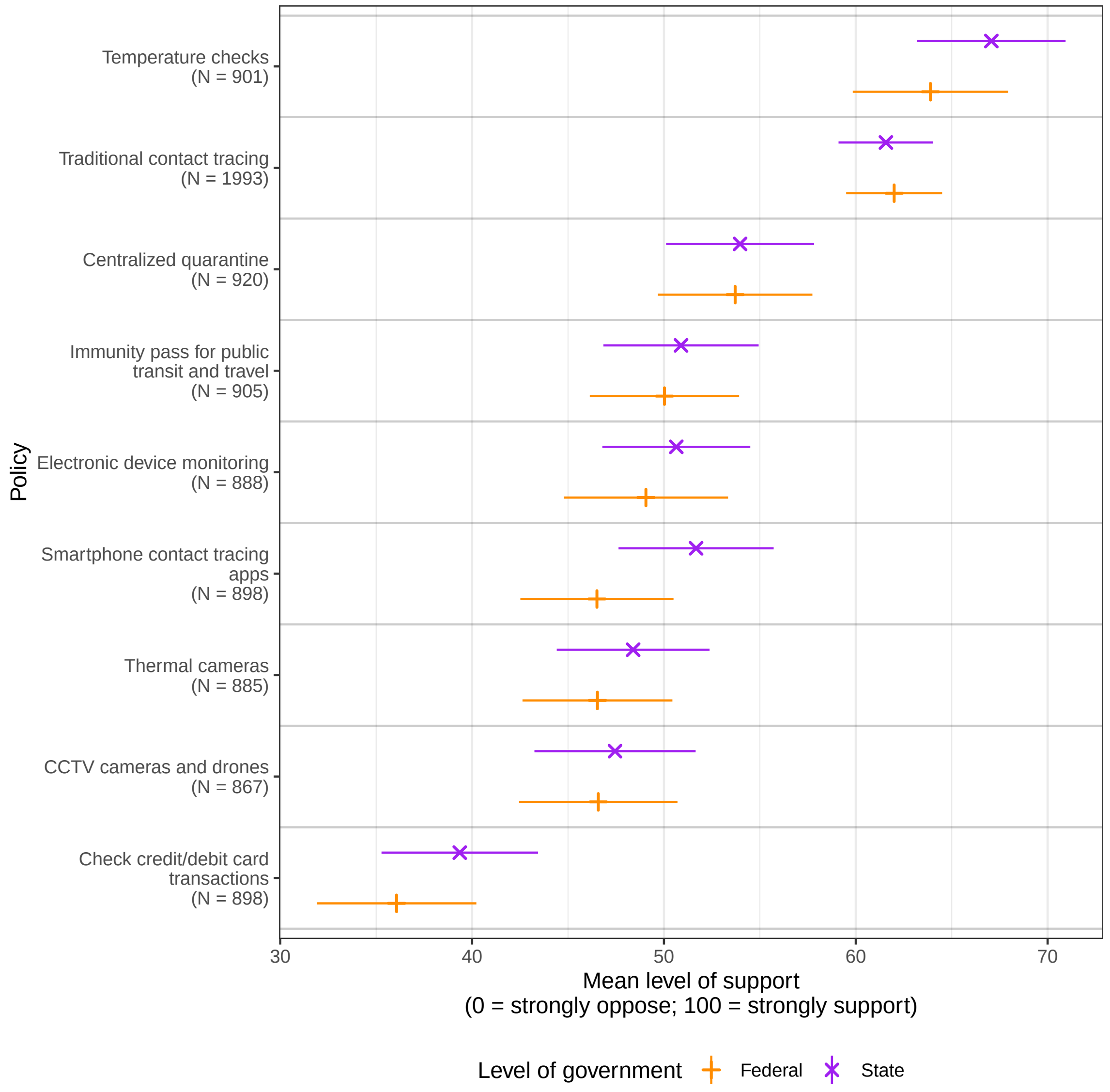

Fig. 4: Mean levels of support for surveillance policies by level of government mentioned with 95% confidence intervals. Level of government (state or federal) is randomly assigned at the level of the respondent.

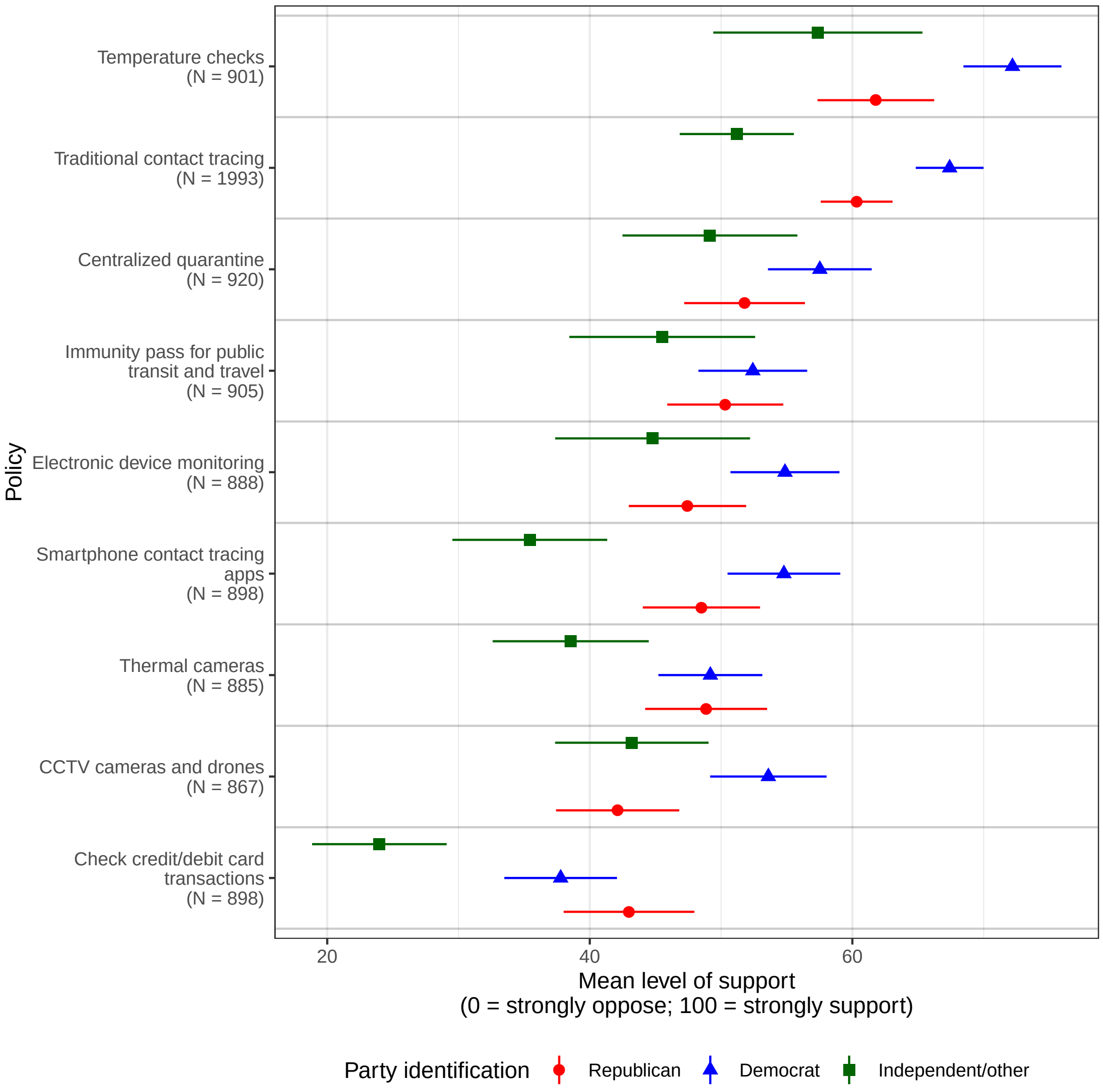

Fig. 5: Mean levels of support for surveillance policies by party identification, with 95% confidence intervals.

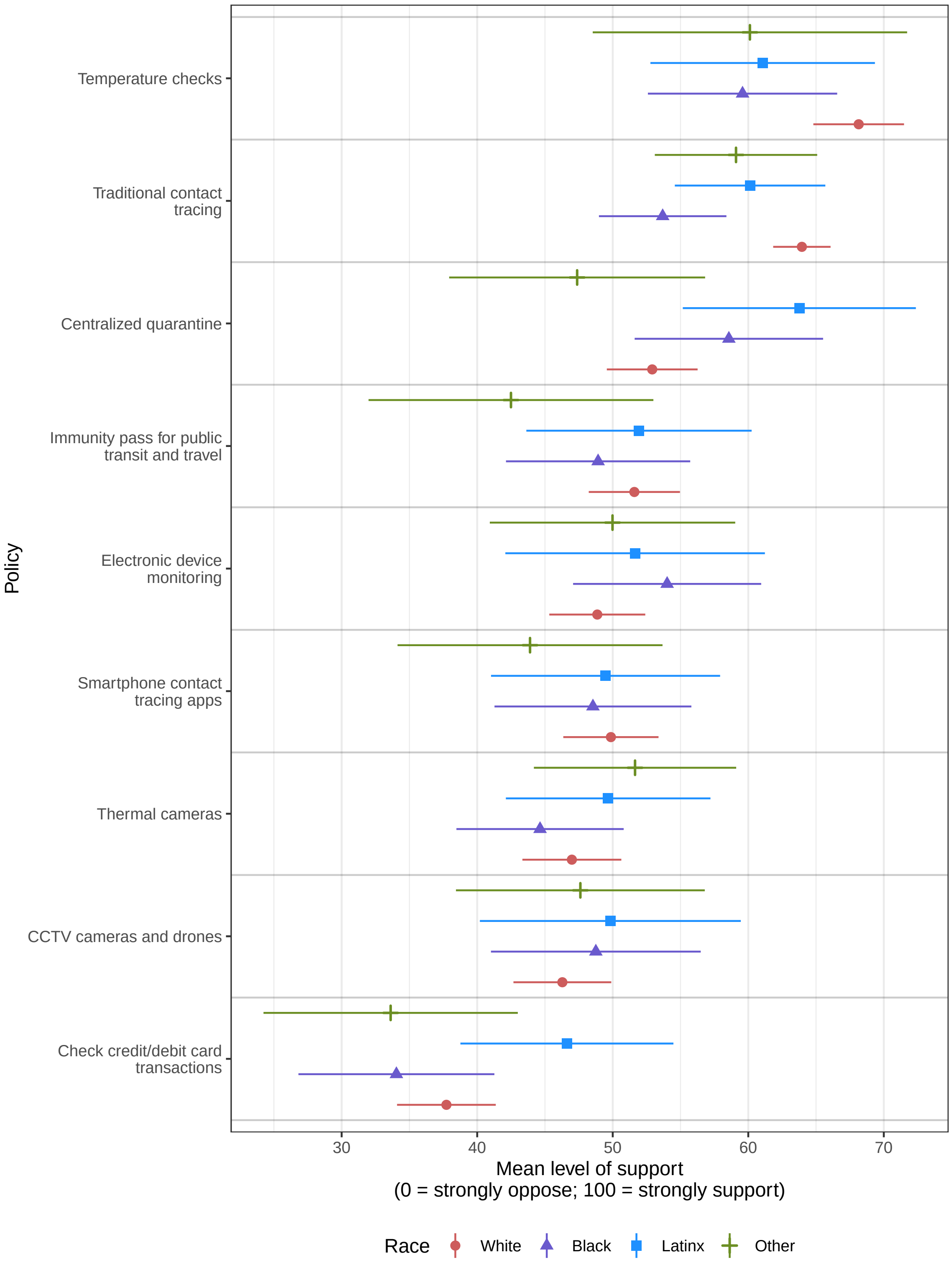

Fig. 6: Mean levels of support for surveillance policies by race, with 95% confidence intervals.

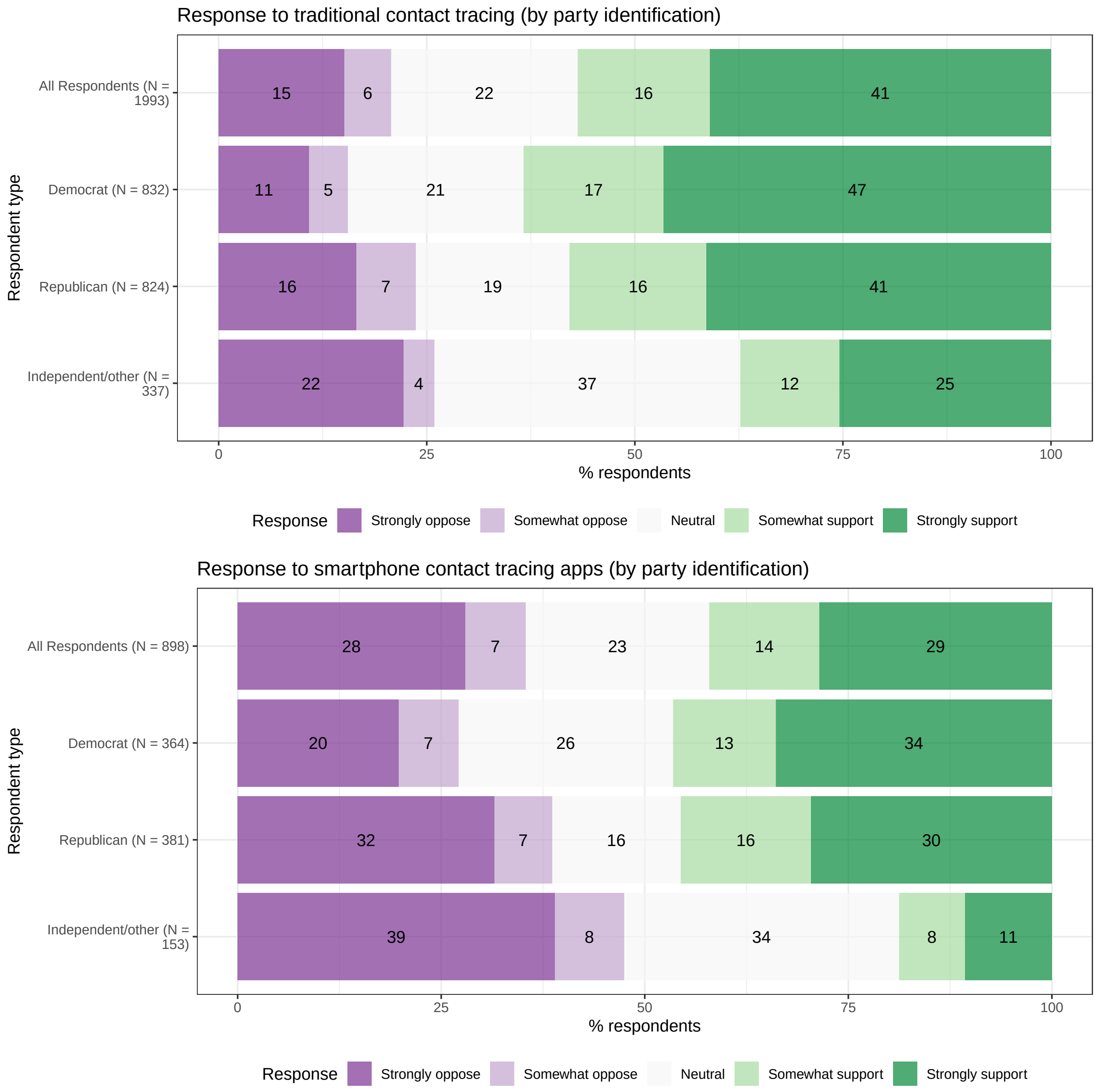

Fig. 7: Distribution of support for government policies expanding traditional contact tracing and encouraging smartphone contact tracing apps use by party identification. Respondents were asked to indicate their support for each policy by entering a number between 0 and 100, where 0 = strongly oppose and 100 = strongly support. Categories shown in the figure divide the raw responses into five categories (0-20 = Strongly oppose, 21-40 = Somewhat oppose, 41-60 = Neutral, 61-80 = Somewhat support, 81-100 = Strongly support).

Table 4: Level of government and support for surveillance.

	Dependent variable:			
	Policy support			
	(1)	(2)	(3)	(4)
Federal	−0.626	−2.990	1.675	−0.141
	(1.145)	(1.618)	(1.882)	(2.519)
Republican		-7.256^{***}		-7.047^{**}
		(1.773)		(2.610)
Federal x Republican		4.866		3.727
		(2.519)		(3.681)
Copartisan			2.282	1.450
			(1.782)	(2.224)
Federal × Copartisan			−4.532	−4.650
			(2.536)	(3.281)
Republican × Copartisan				−0.068
				(3.566)
Federal × Republican × Copartisan				0.930
				(5.070)
Constant	52.189^{***}	57.085^{***}	52.350^{***}	56.190^{***}
	(0.803)	(1.095)	(1.336)	(1.709)
Subset	All	D & R	D & R	D & R
Respondents	2,528	1,952	1,952	1,952
Observations	9,155	7,627	7,627	7,627
R^2	0.0001	0.006	0.001	0.007
Adjusted R^2	−0.00003	0.006	0.001	0.006

Cluster robust standard errors at respondent level $^{*}p<0.05$; $^{**}p<0.01$; $^{***}p<0.001$

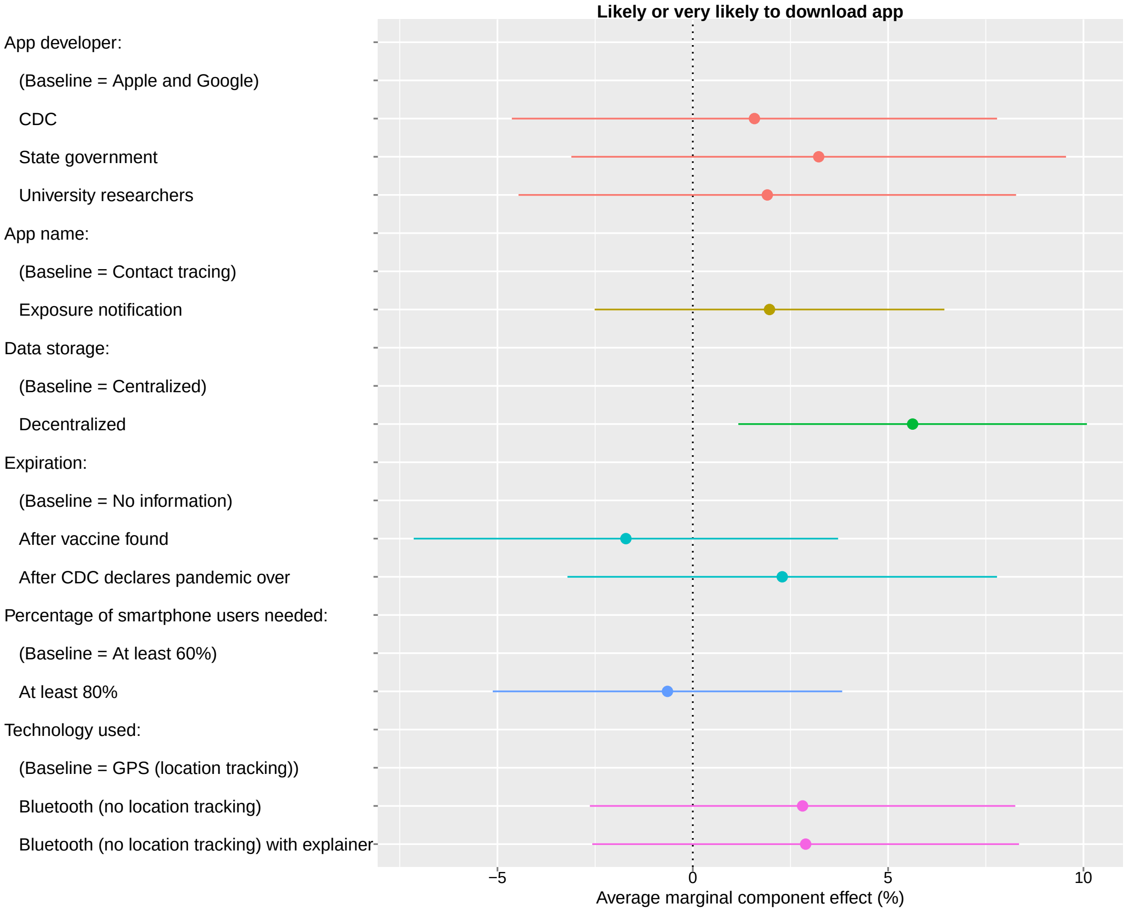

Fig. 8: Average marginal component effects of contacting tracing app attributes on probability a respondent is likely to download the app. Outcome is binary, coded as "likely" (1) if the respondent answered 60 or above on a continuous scale when asked how likely they would be to download the app described, and zero (0) if the respondent entered a number below 60. Question was asked of all respondents who reported owning a smartphone ($N = 1,883$).

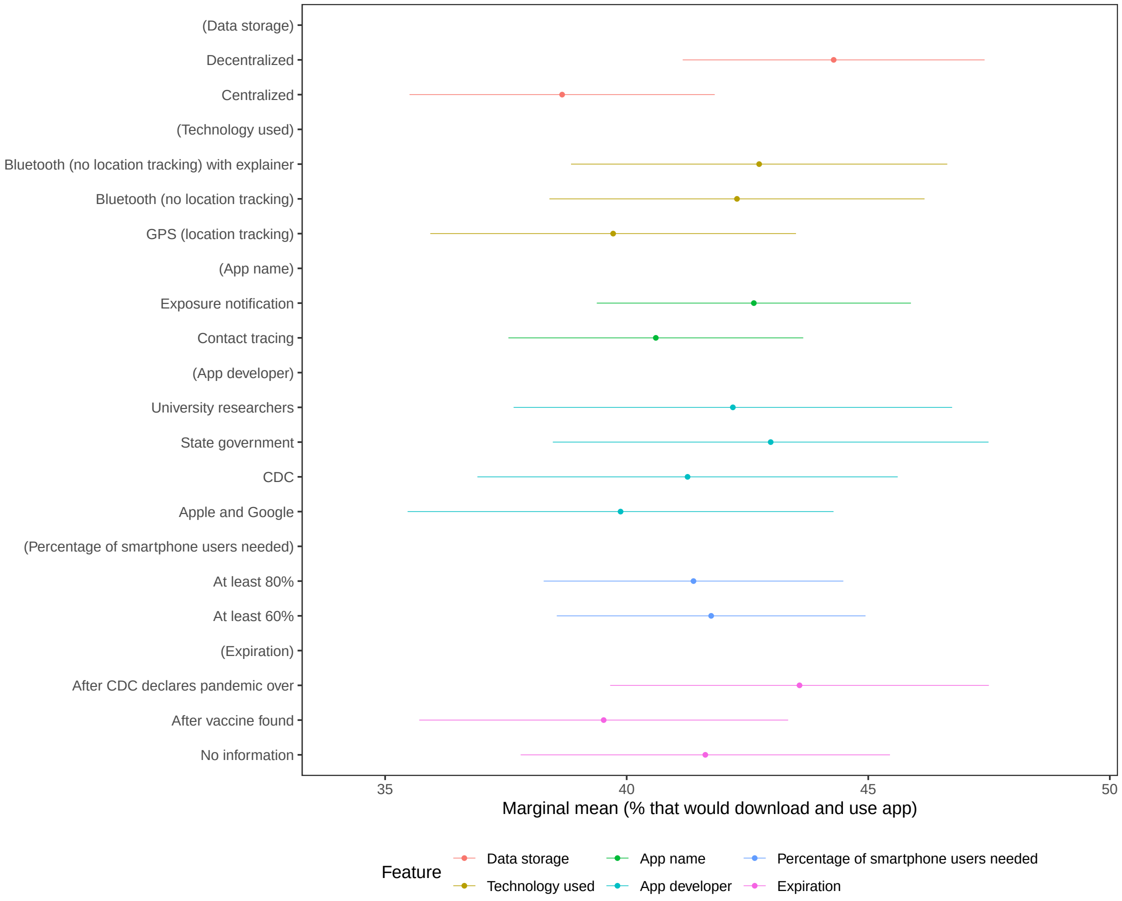

Fig. 9: Marginal mean probability a respondent is likely to download the app for each conjoint attribute value. Outcome is binary, coded as "likely" (1) if the respondent answered 60 or above on a continuous scale when asked how likely they would be to download the app described, and zero (0) if the respondent entered a number below 60. Question was asked of all respondents who reported owning a smartphone ($N = 1,883$)

Table 5: Marginal means from the conjoint analysis: likelihood to download and use app ($N = 1,883$).

Feature type	Feature	Marginal means (likelihood)	SE	Marginal means (percent with >60% likelihood)
Data storage	Centralized	44.09	1.22	38.71
Data storage	Decentralized	49.48	1.21	44.28
Technology used	GPS (location tracking)	44.92	1.49	39.72
Technology used	Bluetooth (no location tracking)	46.73	1.49	42.28
Technology used	Bluetooth (no location tracking) with explainer	49.03	1.49	42.81
App name	Contact tracing	46.34	1.19	40.60
App name	Exposure notification	47.47	1.25	42.68
App developer	Apple and Google	44.44	1.75	39.87
App developer	CDC	47.39	1.66	41.26
App developer	State government	47.93	1.71	42.98
App developer	University researchers	47.76	1.77	42.29
Percentage of smartphone users needed	At least 60%	46.37	1.24	41.75
Percentage of smartphone users needed	At least 80%	47.34	1.20	41.43
Expiration	No information	48.12	1.46	41.63
Expiration	After vaccine found	44.89	1.49	39.52
Expiration	After CDC declares pandemic over	47.59	1.52	43.65

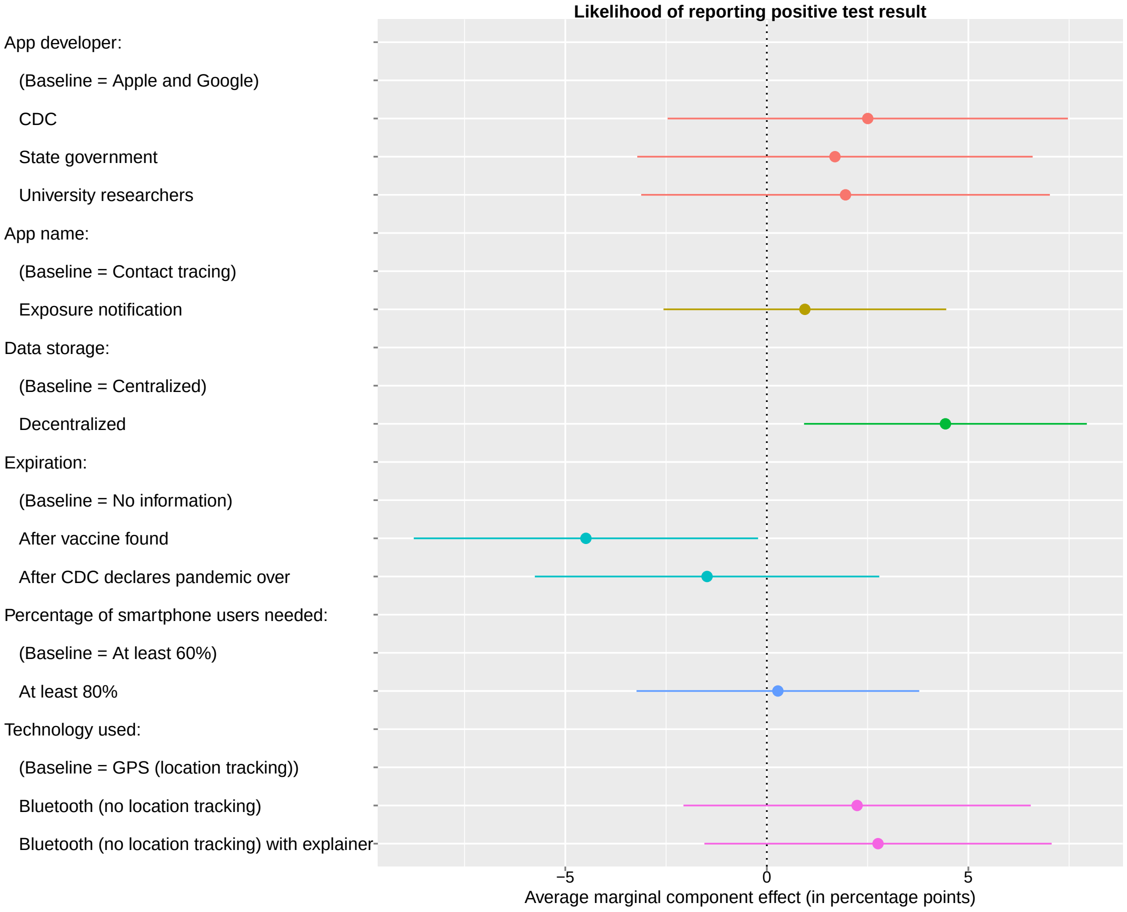

Fig. 10: Average marginal component effects of contacting tracing app attributes on reported likelihood of reporting a positive COVID-19 test to the app with 95% confidence intervals. Outcome is measured using a continuous scale from 0 to 100. Question was asked of all respondents who reported owning a smartphone ($N = 1,883$).

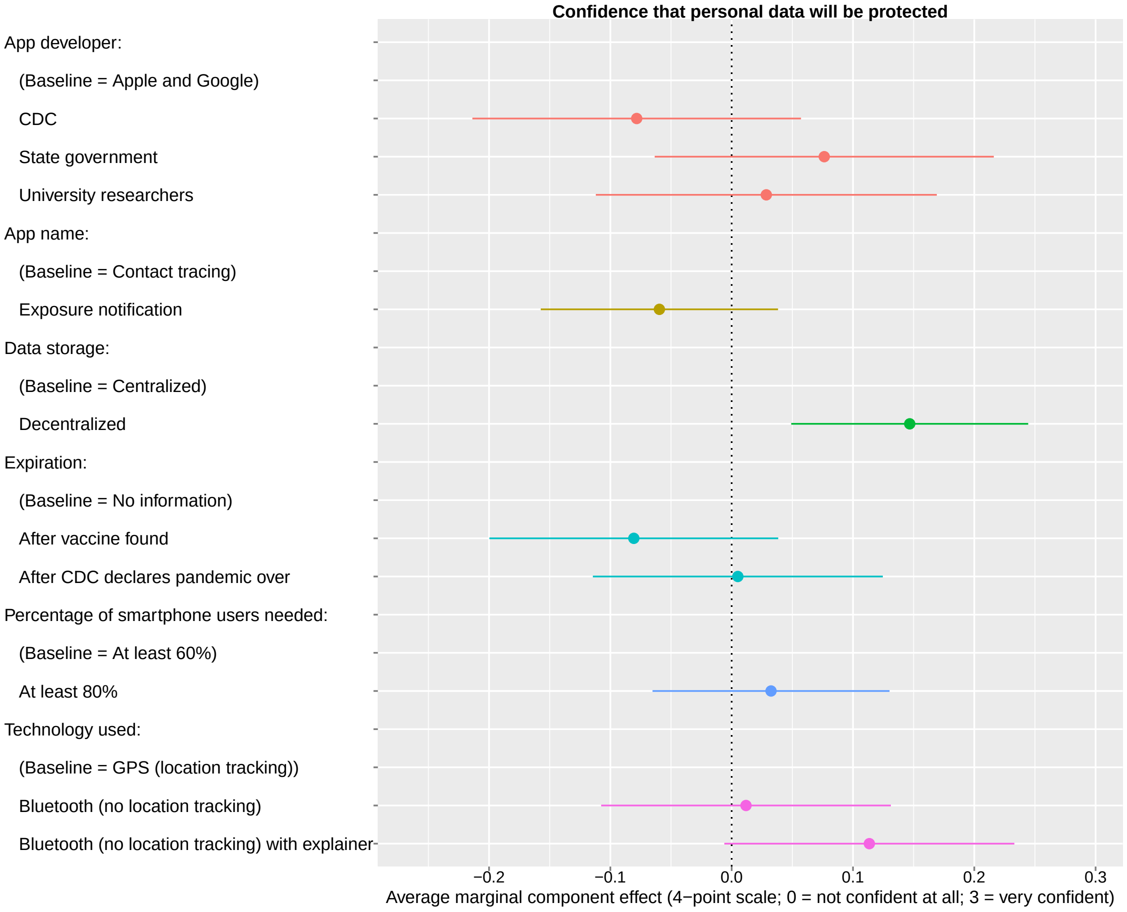

Fig. 11: Average marginal component effects of contacting tracing app attributes on whether respondents perceive that their personal data will be protected, with 95% confidence intervals. Outcome is measured using a 4-point scale, where 0 means "not confident at all" and 3 means "very confident" ($N = 2,057$).

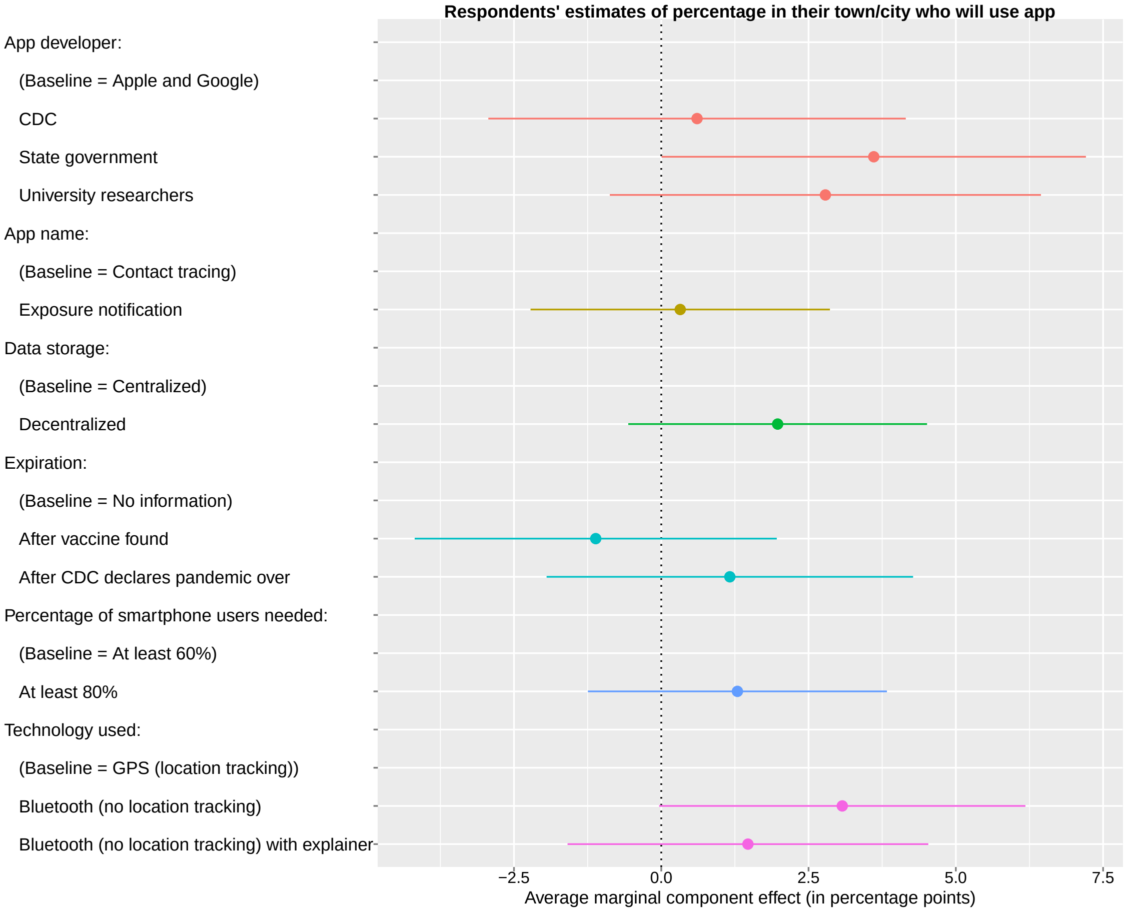

Fig. 12: Average marginal component effects of contacting tracing app attributes on percentage of people in respondents' town or city they believe would be likely to download the app, with 95% confidence intervals. Outcome is measured using a continuous scale from 0 to 100. Question was asked of all respondents ($N = 2,057$)

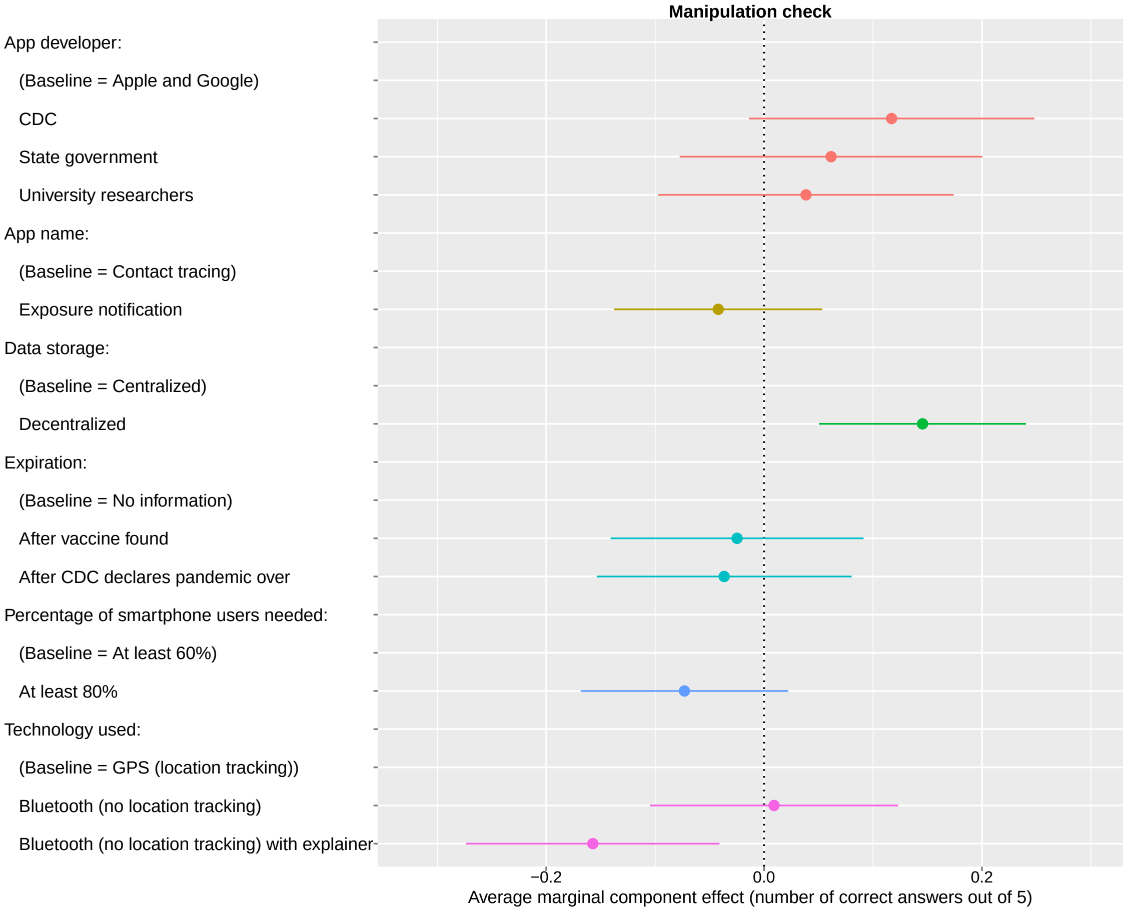

Fig. 13: Average marginal component effects of contacting tracing app attributes on the number of correct answers in the manipulation check, with 95% confidence intervals. Respondents were given five statements and asked to check all the correct ones based on the description of the contact tracing app they read about ($N = 2,057$)

Table 6: Conjoint analysis results: percentage agreement with statement 'The government should require everyone who has a smartphone to use this app.' Respondents who answered "strongly agree" or "somewhat agree" are counted as those who agree. $N = 2,057$.

Feature type	Feature	Prop. agree	SE
Overall	–	0.36	0.01
Data storage	Centralized	0.35	0.02
Data storage	Decentralized	0.37	0.01
Technology used	GPS (location tracking)	0.32	0.02
Technology used	Bluetooth (no location tracking)	0.36	0.02
Technology used	Bluetooth (no location tracking) with explainer	0.39	0.02
App name	Contact tracing	0.35	0.01
App name	Exposure notification	0.36	0.02
App developer	Apple and Google	0.36	0.02
App developer	CDC	0.30	0.02
App developer	State government	0.40	0.02
App developer	University researchers	0.38	0.02
Percentage of smartphone users needed	At least 60%	0.36	0.02
Percentage of smartphone users needed	At least 80%	0.35	0.01
Expiration	No information	0.36	0.02
Expiration	After vaccine found	0.35	0.02
Expiration	After CDC declares pandemic over	0.36	0.02

Table 7: Conjoint analysis results: percentage agreement with statement 'Employers should require their employees with smartphones to use this app.' Respondents who answered "strongly agree" or "somewhat agree" are counted as those who agree. $N = 2,057$.

Feature type	Feature	Prop. agree	SE
Overall	–	0.39	0.01
Data storage	Centralized	0.37	0.02
Data storage	Decentralized	0.40	0.02
Technology used	GPS (location tracking)	0.36	0.02
Technology used	Bluetooth (no location tracking)	0.38	0.02
Technology used	Bluetooth (no location tracking) with explainer	0.42	0.02
App name	Contact tracing	0.39	0.01
App name	Exposure notification	0.38	0.02
App developer	Apple and Google	0.38	0.02
App developer	CDC	0.35	0.02
App developer	State government	0.41	0.02
App developer	University researchers	0.42	0.02
Percentage of smartphone users needed	At least 60%	0.39	0.02
Percentage of smartphone users needed	At least 80%	0.38	0.02
Expiration	No information	0.39	0.02
Expiration	After vaccine found	0.39	0.02
Expiration	After CDC declares pandemic over	0.39	0.02

Table 8: Conjoint analysis results: percentage agreement with statement 'Apple and Google should automatically install this app on users' iPhones or Android phones as part of a software update.' Respondents who answered "strongly agree" or "somewhat agree" are counted as those who agree. $N = 2,057$.

Feature type	Feature	Prop. agree	SE
Overall	–	0.34	0.01
Data storage	Centralized	0.33	0.01
Data storage	Decentralized	0.35	0.01
Technology used	GPS (location tracking)	0.31	0.02
Technology used	Bluetooth (no location tracking)	0.35	0.02
Technology used	Bluetooth (no location tracking) with explainer	0.36	0.02
App name	Contact tracing	0.34	0.01
App name	Exposure notification	0.34	0.02
App developer	Apple and Google	0.34	0.02
App developer	CDC	0.29	0.02
App developer	State government	0.38	0.02
App developer	University researchers	0.35	0.02
Percentage of smartphone users needed	At least 60%	0.33	0.01
Percentage of smartphone users needed	At least 80%	0.35	0.01
Expiration	No information	0.34	0.02
Expiration	After vaccine found	0.32	0.02
Expiration	After CDC declares pandemic over	0.36	0.02

Table 9: Conjoint analysis results: percentage agreement with statement 'Places of religious worship, like churches, synagogues, and mosques, should require everyone who has a smartphone to use this app if they want to worship there.' Respondents who answered "strongly agree" or "somewhat agree" are counted as those who agree. $N = 2,057$.

Feature type	Feature	Prop. agree	SE
Overall	–	0.37	0.01
Data storage	Centralized	0.34	0.02
Data storage	Decentralized	0.40	0.02
Technology used	GPS (location tracking)	0.36	0.02
Technology used	Bluetooth (no location tracking)	0.37	0.02
Technology used	Bluetooth (no location tracking) with explainer	0.39	0.02
App name	Contact tracing	0.36	0.01
App name	Exposure notification	0.38	0.02
App developer	Apple and Google	0.37	0.02
App developer	CDC	0.34	0.02
App developer	State government	0.41	0.02
App developer	University researchers	0.38	0.02
Percentage of smartphone users needed	At least 60%	0.36	0.02
Percentage of smartphone users needed	At least 80%	0.38	0.01
Expiration	No information	0.37	0.02
Expiration	After vaccine found	0.36	0.02
Expiration	After CDC declares pandemic over	0.38	0.02

Table 10: Conjoint analysis results: percentage agreement with statement 'App users should have a choice in sharing their test outcomes with the app if they tested positive for COVID-19.' Respondents who answered "strongly agree" or "somewhat agree" are counted as those who agree. $N = 2,057$.

Feature type	Feature	Prop. agree	SE
Overall	–	0.58	0.01
Data storage	Centralized	0.59	0.02
Data storage	Decentralized	0.58	0.02
Technology used	GPS (location tracking)	0.56	0.02
Technology used	Bluetooth (no location tracking)	0.58	0.02
Technology used	Bluetooth (no location tracking) with explainer	0.60	0.02
App name	Contact tracing	0.58	0.02
App name	Exposure notification	0.58	0.02
App developer	Apple and Google	0.59	0.02
App developer	CDC	0.56	0.02
App developer	State government	0.57	0.02
App developer	University researchers	0.61	0.02
Percentage of smartphone users needed	At least 60%	0.58	0.02
Percentage of smartphone users needed	At least 80%	0.58	0.02
Expiration	No information	0.58	0.02
Expiration	After vaccine found	0.58	0.02
Expiration	After CDC declares pandemic over	0.59	0.02

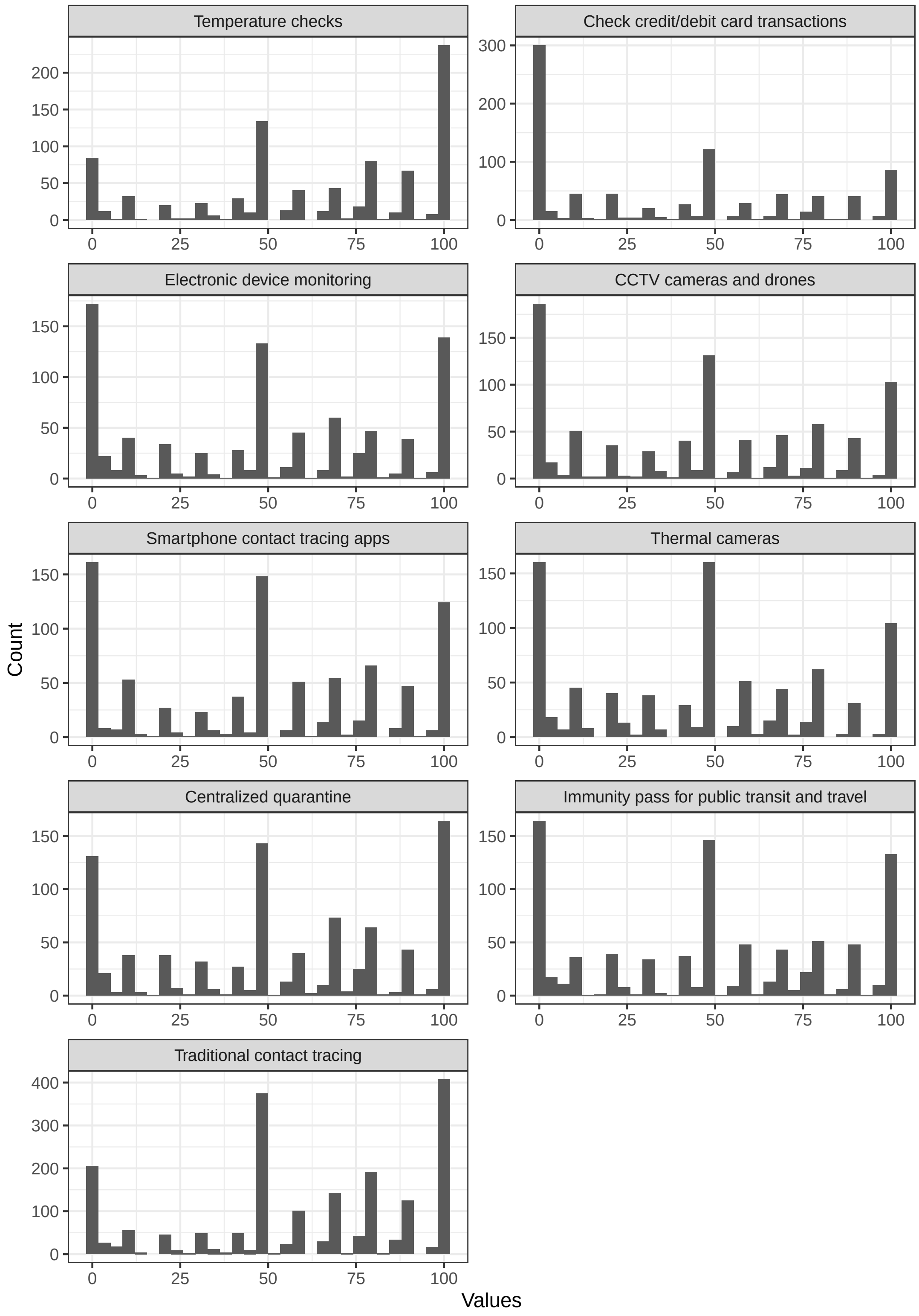

Fig. 14: Distribution of support for public health surveillance policies. Each respondent was asked about the expansion of traditional contact tracing and three other policies, randomly selected. Respondents were asked to express their support for each policy on a scale from 0 to 100, where 0 = strongly oppose and 100 = strongly support.

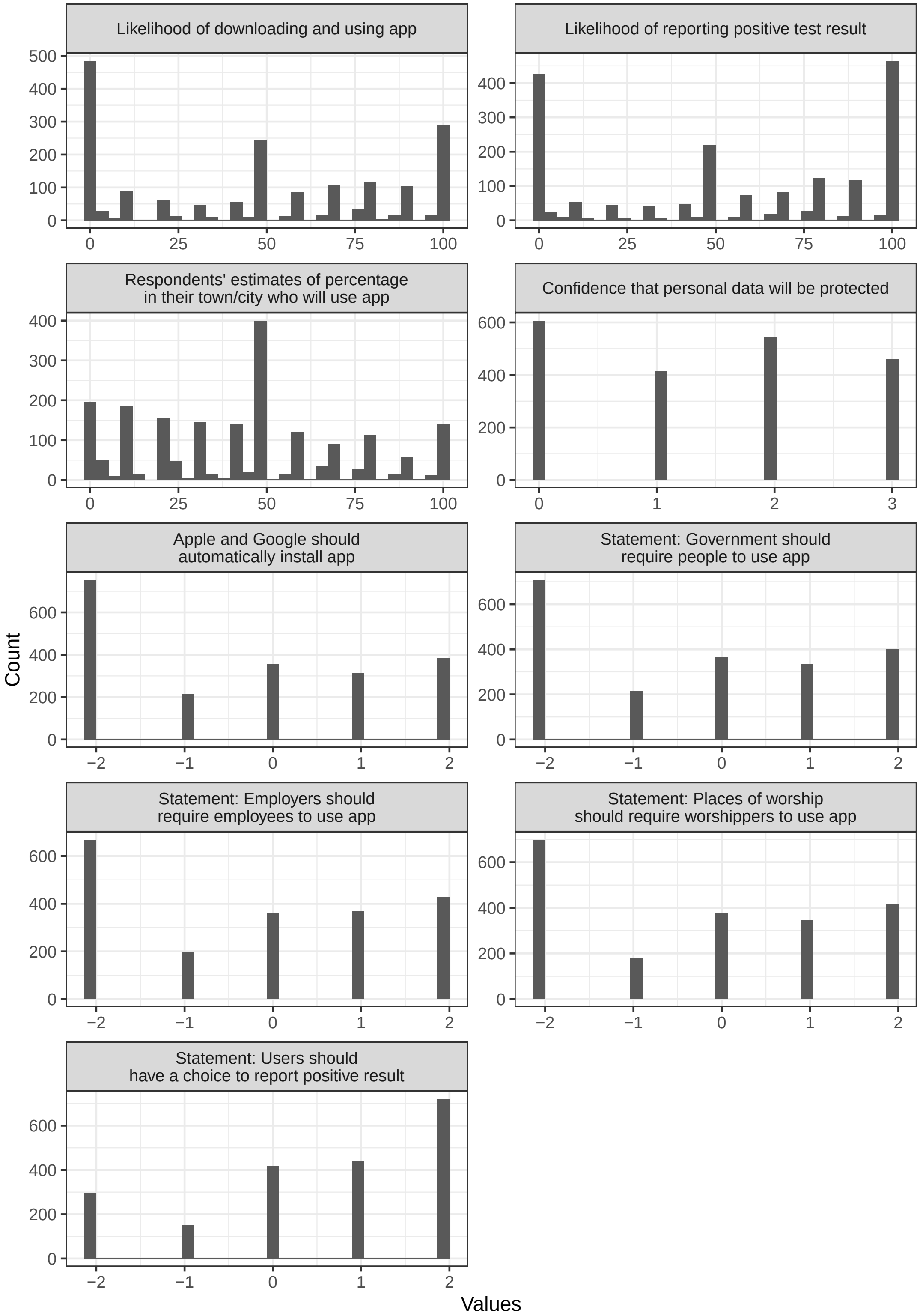

Fig. 15: Distribution of responses to the conjoint analysis questions. Respondents were asked each of these questions after reading a description of a hypothetical contact tracing app. For the first three questions, respondents were asked to answer on a scale from 0 to 100. Respondents were then asked about their confidence their personal data would be protected when using the app (0 indicates "not at all confident" and 3 indicates "very confident). The last four questions asked respondents to indicate their agreement with a provided statement (-2 indicates "strongly disagree" and 2 indicates "strongly agree").

Table 11: Association between trust in institutions and support for surveillance policies. We report results from two regression models where we predict support for the surveillance policies using responses to questions related to trust in institutions or actors. The "mean trust in institutions" variable is the average level of trust respondents have in the three institutions they were randomly assigned to evaluate. Both linear regression models control for the type of policy respondents were asked to evaluate. Model 2 also includes demographic variables including gender, race, party identification, age group, income level, level of education, and geographic region.

	(1)	(2)
(Intercept)	21.773***	18.382***
	(1.785)	(3.414)
Mean trust in institutions	11.117***	9.229***
	(0.861)	(0.887)
Trust in advice related to COVID-19 from Trump	-0.765*	-0.639
	(0.341)	(0.416)
Trust in advice related to COVID-19 from the CDC	4.357***	4.396***
	(0.537)	(0.547)
Trust in advice related to COVID-19 from the respondent's state governor	2.940***	2.665***
	(0.472)	(0.474)
Controls for policy type	Y	Y
Demographic controls	N	Y
Respondents	2,511	2,511
Observations	9,038	9,038
R^2	0.191	0.217
Adjusted R^2	0.190	0.214

CRSE at respondent level
* $p < 0.05$, ** $p < 0.01$, *** $p < 0.001$

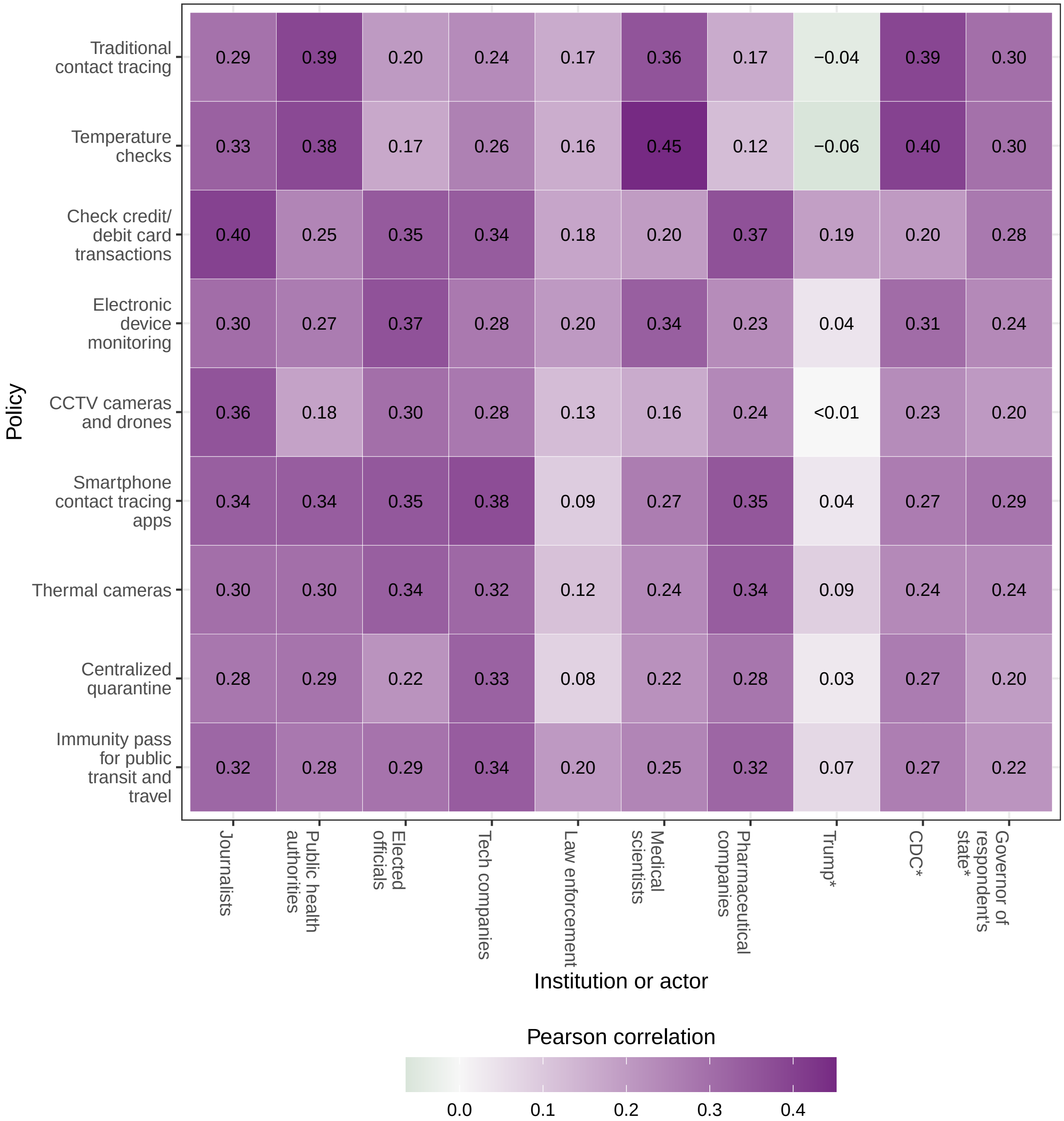

Fig. 16: Pearson correlation between trust in institution or actors and support for surveillance policies. For the institutions or actors that are not marked with an $*$, respondents were presented with three randomly selected actors and asked much confidence they have in each to act in the best interest of the public using a 4-point scale (0 = no confidence at all; 3 = a great deal of confidence). For the institution or actors marked with an $*$, respondents were asked how much they trust advice related to COVID-19 coming from that institution or actors using a 5-point scale (-2 = distrust a lot, 2 = trust a lot); respondents had to evaluate all three institution or actors.

Table 12: Predicting the number of wrong answers in the conjoint analysis manipulation check using support for public health surveillance policies and demographic variables. To check that respondents understood the hypothetical contact tracing app described, respondents were represented with five statements about the app and asked to select all the true statements. The "mean support for public health surveillance policies" variable averages each respondents' support for the public health surveillance policies that they were assigned to evaluate.

	Coefficient (SE)
(Intercept)	2.080***
	(0.161)
Mean support for public health surveillance policies	0.002**
	(0.001)
Female	-0.150**
	(0.050)
Black	-0.023
	(0.080)
Latinx	-0.023
	(0.097)
Other race	0.173*
	(0.081)
Democrat	-0.045
	(0.075)
Republican	0.057
	(0.076)
30-44	-0.001
	(0.074)
45-59	-0.156*
	(0.075)
60+	-0.089
	(0.075)
$50k-99k	-0.037
	(0.061)
>99$k	0.180*
	(0.076)
HS graduate	-0.194
	(0.134)
Some college	-0.444***
	(0.134)
Bachelors or higher	-0.287*
	(0.135)
Northeast	-0.056
	(0.077)
South	-0.069
	(0.066)
West	-0.020
	(0.076)
R^2	0.041
Adjusted R^2	0.032
N	2,057

* $p < 0.05$, ** $p < 0.01$, *** $p < 0.001$

Table 13: Association between outcomes in the conjoint analysis experiments and the number of wrong answers in the manipulation check. For each outcome, Model 1 does not include demographic controls (gender, race, party identification, age group, income level, level of education, and geographic region) while Model 2 does.

DV: Likelihood of downloading and use the contact tracing app	(1)	(2)
(Intercept)	42.358***	30.945***
	(1.624)	(5.363)
Number of wrong answers in the manipulation check	2.541**	1.074
	(0.776)	(0.729)
Demographic controls	N	Y
R^2	0.006	0.138
Adjusted R^2	0.005	0.130
N	1,883	1,883

* p < 0.05, ** p < 0.01, *** p < 0.001

DV: Likelihood of reporting positive test result	(1)	(2)
(Intercept)	52.607***	34.855***
	(1.698)	(5.731)
Number of wrong answers in the manipulation check	0.933	0.300
	(0.789)	(0.775)
Demographic controls	N	Y
R^2	0.001	0.081
Adjusted R^2	< 0.001	0.072
N	1,883	1,883

* p < 0.05, ** p < 0.01, *** p < 0.001

DV: Response to statement: Government should require smartphone users to download and use the app	(1)	(2)
(Intercept)	-0.664***	-0.102
	(0.063)	(0.205)
Number of wrong answers in the manipulation check	0.237***	0.156***
	(0.031)	(0.028)
Demographic controls	N	Y
R^2	0.029	0.199
Adjusted R^2	0.029	0.192
N	2,057	2,057

* p < 0.05, ** p < 0.01, *** p < 0.001

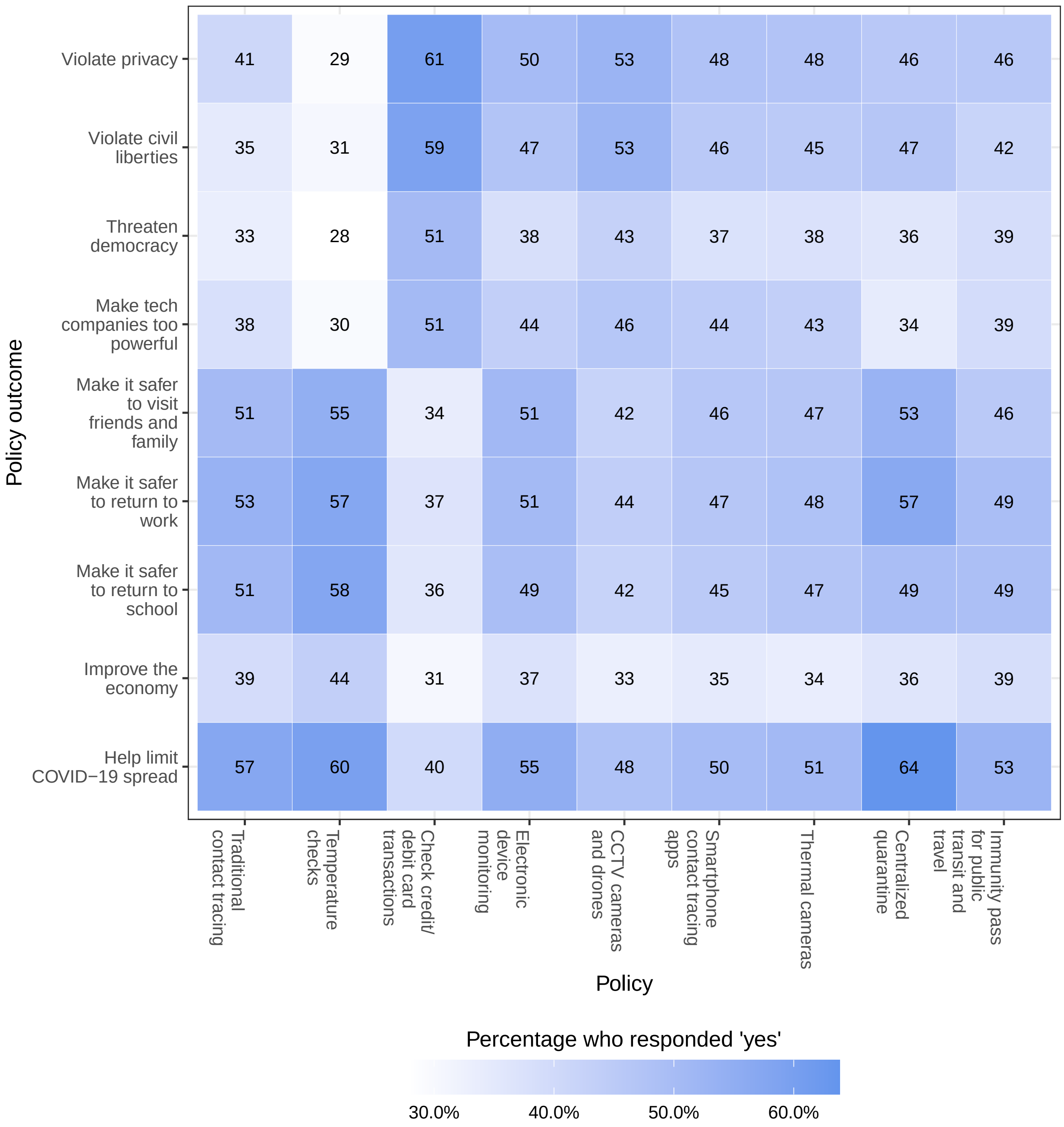

Fig. 17: Respondents' predicted outcomes of the surveillance policies. For each surveillance policy the respondent read about, they were presented with nine outcomes (the order was randomized) and asked if they think each outcome would happen if the policy were adopted. The answer choices included "yes," "no," and "don't know." The figure presents the percentage of respondents who answered "yes" for each policy and outcome.

Table 14: Proportion of correct responses to each statement in the conjoint analysis manipulation check. Respondents were presented with the following five statements such that the order of the statements were randomized. They were asked to select all the statements that are true of the hypothetical contact tracing app that they read about. Respondents could refer to the description of the app (available as collapsible content) while answering the manipulation check question.

Statement	Proportion answered correctly
The app will track your location data.	0.65
The app will send you the names of infected people you have been in close contact with.	0.69
All user data will be stored on a central server.	0.45
Apple and Google are building this app.	0.77
Public health experts say that at least 60% of smartphone users needs to use this app for it to be effective at limiting the spread of COVID-19.	0.67
