# Supplementary material for: Americans’ perceptions of privacy and surveillance in the COVID-19 pandemic
Source: PLoS One. 2020 Dec 23;15(12):e0242652. doi: 10.1371/journal.pone.0242652 (PMC7757814; doi:10.1371/journal.pone.0242652)
Supplement: S2 File — The survey instrument file provides detailed information about the questions, branching conditions, and consent options presented to respondents. (PDF) [file pone.0242652.s002.pdf]

Survey Instrument

Americans' perceptions of privacy and surveillance in the COVID-19 Pandemic
Survey Instrument

Baobao Zhang, Sarah Kreps, Nina McMurry, and R. Miles McCain

November 18, 2020

COVID Privacy Study Survey 2

Survey Flow

EmbeddedData
ridValue will be set from Panel or URL.
ageValue will be set from Panel or URL.
genderValue will be set from Panel or URL.
hhiValue will be set from Panel or URL.
ethnicityValue will be set from Panel or URL.
hispanicValue will be set from Panel or URL.
educationValue will be set from Panel or URL.
political_partyValue will be set from Panel or URL.
regionValue will be set from Panel or URL.
zipValue will be set from Panel or URL.
UserAgentValue will be set from Panel or URL.

EmbeddedData
proceed = 1
explainer_graphic = ${rand://int/0:2}
thoughts_app = ${rand://int/1:5}
clicked = 0
textbox_policy = 0
vaccine = ${rand://int/0:1}

BlockRandomizer: 1 -

EmbeddedData
government_level = your state government
government_level_q = Your state government

EmbeddedData
government_level = the federal government
government_level_q = The federal government

BlockRandomizer: 1 -

EmbeddedData
app_name = an exposure notification

EmbeddedData
app_name = a contact tracing

BlockRandomizer: 1 -

EmbeddedData
app_developer = Apple and Google are

EmbeddedData
app_developer = The Centers for Disease Control and Prevention (CDC) is

EmbeddedData
app_developer = Your state government is

EmbeddedData
app_developer = A group of researchers at leading universities is

Branch: New Branch
If
If explainer_graphic Is Not Equal to 1

BlockRandomizer: 1 -

EmbeddedData
tech_used = GPS data that track users’ location

EmbeddedData

tech_used = Bluetooth data that does not track users’ location

BlockRandomizer: 1 -

EmbeddedData
percent_needed = 60
EmbeddedData
percent_needed = 80

BlockRandomizer: 1 -

EmbeddedData
data_storage = All user data will be stored on users’ phones. A central server will be used only to relay information between users only when necessary, like notifying someone if they have been in close contact with a person who has tested positive.
costs_benefits = The decentralized data storage makes users’ data more protected against intrusions by hackers or the government. However, this setup would give public health authorities less ability to manage and analyze user data.
actual_use = By storing data on users' phones, the app can quietly run in the background. Users do not have to unlock their phones and have the app open for the app to work.
EmbeddedData
data_storage = All user data will be stored on a central server controlled by the app developer.
costs_benefits = The centralized data storage would give public health authorities greater ability to manage and analyze user data. However, this setup makes users’ data less protected against intrusions by hackers or the government.
actual_use = By storing data on a central server, users must unlock their phones and have the app open for the app to work. While the app is running, users cannot make/take calls or use other apps.

BlockRandomizer: 1 -

EmbeddedData
expire_app = The app would expire after the Centers for Disease Control and Prevention (CDC) declares the COVID-19 Pandemic is over.
EmbeddedData
expire_app = The app would expire after a successful vaccine for COVID-19 has been discovered.
EmbeddedData
expire_appValue will be set from Panel or URL.

BlockRandomizer: 1 -

EmbeddedData
experience_prime = 1
EmbeddedData
experience_prime = 0

BlockRandomizer: 1 -

EmbeddedData
notification_download = Suppose that you got a notification on your phone to download this app.
EmbeddedData
notification_downloadValue will be set from Panel or URL.

Standard: IRB Consent (2 Questions)

Branch: New Branch
If

If Welcome! You are being asked to participate in a research study conducted by researchers at the M... I do not agree to participate Is Selected

EndSurvey: Advanced

Standard: COVID-19 Response (13 Questions)

Branch: New Branch
If
If experience_prime Is Equal to 1

Standard: COVID-19 Experience (18 Questions)

Branch: New Branch
If
If thoughts_app Is Not Equal to 1

Standard: Traditional Contact Tracing (5 Questions)
Standard: Limits of Acceptable Surveillance Intro (1 Question)

Branch: New Branch
If
If thoughts_app Is Equal to 1

Standard: Limits of Acceptable Surveillance Intro - No TCT (1 Question)

Standard: Limits of Acceptable Surveillance (5 Questions)
Standard: Conjoint Analysis (6 Questions)

EmbeddedData
ca_nographic = ${q://QID96/QuestionText}
ca_graphic = ${q://QID93/QuestionText}

Standard: Conjoint analysis reading check and reflection (9 Questions)
Standard: Conjoint analysis main questions (9 Questions)
Standard: Conjoint analysis agreement block (8 Questions)
Standard: Vaccine acceptance (3 Questions)
Block: Demographics (28 Questions)
Standard: Final (3 Questions)

Branch: New Branch
If
If experience_prime Is Equal to 0

Branch: New Branch
If
If thoughts_app Is Not Equal to 1

Standard: Traditional Contact Tracing (5 Questions)
Standard: Limits of Acceptable Surveillance Intro (1 Question)

Branch: New Branch
If
If thoughts_app Is Equal to 1

Standard: Limits of Acceptable Surveillance Intro - No TCT (1 Question)

Standard: Limits of Acceptable Surveillance (5 Questions)

Standard: Conjoint Analysis (6 Questions)

EmbeddedData
ca_nographic = ${q://QID96/QuestionText}
ca_graphic = ${q://QID93/QuestionText}

Standard: Conjoint analysis reading check and reflection (9 Questions)
Standard: Conjoint analysis main questions (9 Questions)
Standard: Conjoint analysis agreement block (8 Questions)
Standard: COVID-19 Experience (18 Questions)
Standard: Vaccine acceptance (3 Questions)
Block: Demographics (28 Questions)
Standard: Final (3 Questions)

EndSurvey: Advanced

Page Break

Start of Block: IRB Consent

consent
Welcome!

You are being asked to participate in a research study conducted by researchers at the Massachusetts Institute of Technology and Cornell University about important topics in the news today.
Your participation is voluntary. If you decide to participate, you may withdraw at any point during the survey for any reason before submitting your answers by closing the browser.

If you complete the survey, your answers will be recorded and used for research purposes. No identifying information about you will be made public and any views you express will be kept completely confidential.

Findings from this study may be reported in scholarly journals, at academic seminars, and research association meetings, and shared with policymakers.

Lucid is the data controller with respect to your personal data and, as such, will determine how your personal data is used. Please see their privacy notice here: https://luc.id/privacy-policy/. Lucid will share only fully anonymized data with the researchers, for the purposes of research.

The anonymized data will be stored in a secure location and retained indefinitely. If you have any questions, you may contact Baobao Zhang at zhangb@mit.edu.

Please select one of the following options. If you choose not to participate, the survey will end immediately.

- ○ I agree to participate (1)
- ○ I do not agree to participate (0)

consent_t Timing
First Click (1)
Last Click (2)
Page Submit (3)
Click Count (4)

End of Block: IRB Consent

Start of Block: COVID-19 Response

covid_response_intro The following questions relate to your opinions on responses to the COVID-19 pandemic.

Page Break

covid_news On average, how much time per day do you spend reading or listening to news about the COVID-19 pandemic?

- Less than 30 minutes (1)
- 30 minutes - 1 hour (2)
- 1 - 2 hours (3)
- More than 2 hours (4)

Page Break

eval_gov_handling Do you approve or disapprove of the **federal government's** handling of the COVID-19 pandemic so far?

- Strongly approve (2)
- Somewhat approve (1)
- Somewhat disapprove (-1)
- Strongly disapprove (-2)

eval_state_handling Do you approve or disapprove of your **state government's** handling of the COVID-19 pandemic so far?

- Strongly approve (2)
- Somewhat approve (1)
- Somewhat disapprove (-1)
- Strongly disapprove (-2)

Page Break

worst_over Thinking about the COVID-19 pandemic, do you think the worst is over or the worst is yet to come?

- The worst is over (1)
- The worst is yet to come (2)
- Not sure (3)

Page Break

measures_fed Overall, do you think that the **federal government**'s measures taken to control the virus so far have been reasonable, too strict, or not strict enough?

- Reasonable (1)
- Too strict (2)
- Not strict enough (3)

---

measures_state Overall, do you think that **your state government**'s measures taken to control the virus so far have been reasonable, too strict, or not strict enough?

- Reasonable (1)
- Too strict (2)
- Not strict enough (3)

Page Break

trust_advice_people How much do you trust advice related to the COVID-19 pandemic from the following people or organizations?

	Trust a lot (2)	Trust somewhat (1)	Neither trust nor distrust (0)	Distrust somewhat (-1)	Distrust a lot (-2)
Donald Trump (1)	○	○	○	○	○
Centers for Disease Control and Prevention (CDC) (6)	○	○	○	○	○
Your state's governor (9)	○	○	○	○	○

Page Break

stay_con_rights Do you believe that stay-at-home/shelter-in-place orders are a violation of constitutional rights?

- Yes (1)
- No (2)
- Not sure (3)

Page Break

concern_info How concerned are you, if at all, about how much personal information the following people or groups might know about you?

	Very concerned (3)	Somewhat concerned (2)	Not too concerned (1)	Not at all concerned (0)
Tech companies (concern_info_1)	○	○	○	○
The federal government (concern_info_2)	○	○	○	○
Law enforcement (concern_info_3)	○	○	○	○
Public health authorities (concern_info_4)	○	○	○	○

---

confident_cos_t Timing
First Click (1)
Last Click (2)
Page Submit (3)
Click Count (4)

Page Break

trust_inst How much confidence, if any, do you have in each of the following to act in the best interests of the public?

	A great deal of confidence (1)	A fair amount of confidence (6)	Not too much confidence (7)	No confidence at all (5)
Journalists (15)	○	○	○	○
Public health authorities (16)	○	○	○	○
Elected officials (17)	○	○	○	○
Tech companies (18)	○	○	○	○
Law enforcement (19)	○	○	○	○
Medical scientists (20)	○	○	○	○
Pharmaceutical companies (21)	○	○	○	○

---

trust_inst_t Timing
First Click (1)
Last Click (2)
Page Submit (3)
Click Count (4)

End of Block: COVID-19 Response

Start of Block: COVID-19 Experience

affectjob Have you or someone else in your household lost your job or had your hours reduced as a result of the COVID-19 pandemic?

○ Yes (1)

○ No (0)

---

affectjob_t Timing
First Click (1)
Last Click (2)
Page Submit (3)
Click Count (4)

Page Break

Display This Question:

If affectjob = 0

affectjob_likely How likely do you think it is that you or someone else in your household will experience a loss of or reduction in your hours as a result of the COVID-19 pandemic?

- Very likely (3)
- Somewhat likely (2)
- Not very likely (1)
- Not at all likely (0)

---

affectjob_likely_t Timing
First Click (1)
Last Click (2)
Page Submit (3)
Click Count (4)

Page Break

school_work_home Are you currently going to work/school or staying home from work/school?

- Going to work or school (1)
- Staying home from work or school (2)
- I am currently not working or going to school (3)

---

school_work_home_t Timing
First Click (1)
Last Click (2)
Page Submit (3)
Click Count (4)

Page Break

financial_change Would you say that your personal financial situation is improving, getting worse, or staying the same?

- Improving (1)
- Staying the same (0)
- Getting worse (-1)

---

financial_change_t Timing
First Click (1)
Last Click (2)
Page Submit (3)
Click Count (4)

Page Break

concern_economy How concerned are you about the effects of COVID-19 on the U.S. economy?

- Very concerned (3)
- Somewhat concerned (2)
- Not very concerned (1)
- Not at all concerned (0)

---

concern_economy_t Timing
First Click (1)
Last Click (2)
Page Submit (3)
Click Count (4)

Page Break

know_test_pos Do you personally know anyone who has tested positive for COVID-19 (including yourself)?

- Yes (1)
- No (0)
- Prefer not to say (-77)

---

know_test_pos_t Timing
First Click (1)
Last Click (2)
Page Submit (3)
Click Count (4)

Page Break

Display This Question:

If know_test_pos = 1

test_positive_who Who has tested positive? Select all that apply.

- ▢ You (1)
- ▢ Immediate family member(s) (2)
- ▢ More distant family member(s) (3)
- ▢ Close friends(s) (4)
- ▢ More distant friend(s) or acquaintance(s) (5)
- ▢ Prefer not to say (6)

---

test_positive_t Timing
First Click (1)
Last Click (2)
Page Submit (3)
Click Count (4)

Page Break

will_infected How likely do you think it is that you, someone in your family, or someone else you personally know will become infected with COVID-19 in the next few weeks?

- Very likely (3)
- Somewhat likely (2)
- Not very likely (1)
- Not at all likely (0)

---

will_infected_t Timing
First Click (1)
Last Click (2)
Page Submit (3)
Click Count (4)

Page Break

know_die Do you personally know anyone who has died due to complications from COVID-19?

- Yes (1)
- No (0)
- Prefer not to say (-77)

---

know_die_t Timing
First Click (1)
Last Click (2)
Page Submit (3)
Click Count (4)

End of Block: COVID-19 Experience

Start of Block: Traditional Contact Tracing

trad_ct_desc In **traditional contact tracing**, public health workers call those who tested positive for COVID-19 and ask them to list the people they had close contact with in the period during which they might have been infectious.

The public health workers then notify those who have had close contact with an infected person (without revealing the identity of the infected person) and ask the contacts to isolate. Several states are currently using this method of contact tracing.

---

*

trad_ct_support **On a 0 to 100 scale, how much do you support or oppose ${e://Field/government_level} expanding traditional contact tracing?**

Input a number below. 0 means strongly oppose; 100 means strongly support.

____

---

trad_ct_t Timing
First Click (1)
Last Click (2)
Page Submit (3)
Click Count (4)

Page Break

trad_ct_effects **If ${e://Field/government_level} were to expand traditional contact tracing, do you think it would...**

	Yes (1)	No (0)	Don't know (-88)
Help limit the spread of COVID-19 (1)	○	○	○
Improve the economy (2)	○	○	○
Make it safer for workers to return to work (3)	○	○	○
Make it safer for students to return to school (4)	○	○	○
Make it safer for me to visit friends and family (5)	○	○	○
Violate people's privacy (6)	○	○	○
Violate people's civil liberties (7)	○	○	○
Threaten US democracy (8)	○	○	○
Make tech companies too powerful (9)	○	○	○

---

trad_ct_t_2 Timing
First Click (1)
Last Click (2)
Page Submit (3)
Click Count (4)

End of Block: Traditional Contact Tracing

Start of Block: Limits of Acceptable Surveillance Intro

limits_intro Next, we want to understand your views of other public health measures to supplement traditional contact tracing and limit the spread of COVID-19 as stay-at-home orders are lifted. You will read about **three policies** that have been adopted or considered for adoption in other countries.

End of Block: Limits of Acceptable Surveillance Intro

Start of Block: Limits of Acceptable Surveillance Intro - No TCT

limits_intro_no_tct Next, we want to understand your views of various public health measures to limit the spread of COVID-19 as stay-at-home orders are lifted. You will read about three policies that have been adopted or considered for adoption in other countries.

End of Block: Limits of Acceptable Surveillance Intro - No TCT

Start of Block: Limits of Acceptable Surveillance

JS

policy_description Consider the following policy:

${lm://Field/1}

policy_support **On a 0 to 100 scale, how much do you support or oppose ${e://Field/government_level} adopting the policy described above?**

Input a number below. 0 means strongly oppose; 100 means strongly support.

____

policy_effects **If ${e://Field/government_level} were to adopt the policy described above, do you think it would...**

	Yes (1)	No (0)	Don't know (-88)
Help limit the spread of COVID-19 (1)	○	○	○
Improve the economy (2)	○	○	○
Make it safer for workers to return to work (3)	○	○	○
Make it safer for students to return to school (4)	○	○	○
Make it safer for me to visit friends and family (5)	○	○	○
Violate people's privacy (6)	○	○	○
Violate people's civil liberties (7)	○	○	○
Threaten US democracy (8)	○	○	○
Make tech companies too powerful (9)	○	○	○

open_ended If you have further thoughts about the policy described above, please write it in the textbox below. Consider these questions: What are some potential benefits of adopting this policy? What are some potential downsides of adopting this policy?

____

____

____

____

____

policy_t Timing
First Click (1)
Last Click (2)
Page Submit (3)
Click Count (4)

End of Block: Limits of Acceptable Surveillance

Start of Block: Conjoint Analysis

X→

cellphone Do you have a cellphone?

◯ Yes (1)

◯ No (0)

Display This Question:

If cellphone = 1

cellphone_type Which of the following best describes the type of cellphone you have?

◯ Android (1)

◯ iPhone (2)

◯ Windows (3)

◯ Blackberry (4)

◯ Basic non-smartphone cellphone (e.g., a flip-phone) (5)

◯ Other (6) ____

Page Break

ca_intro Public health authorities are considered using smartphone apps as a tool to supplement traditional contact tracing. You will read about one such potential app. We'd like your views on this app. Please take at least 30 seconds to read the description of the app below. You will be asked to recall some facts about the app.

Display This Question:

If explainer_graphic = 1

ca_graphic ${e://Field/app_developer} building ${e://Field/app_name} app for smartphones. It will be used to identify and alert people who have come into contact with those infected with COVID-19. The app will recommend those who came into contact with infected persons to self-isolate for two weeks. The app uses Bluetooth data that does not track users' locations. The app will not reveal the identity of infected persons. Here is how the app works:

${e://Field/data_storage}
${e://Field/costs_benefits}

${e://Field/actual_use}

Public health experts say that at least ${e://Field/percent_needed}% of US smartphone users need to use this app for it to be effective at limiting the spread of COVID-19.
${e://Field/expire_app}

Display This Question:

If explainer_graphic != 1

ca_n_graphic ${e://Field/app_developer} building ${e://Field/app_name} app for smartphones. It will be used to identify and alert people who have come into contact with those infected with COVID-19. The app will recommend those who came into contact with infected persons to self-isolate for two weeks. The app uses ${e://Field/tech_used}. The app will not reveal the identity of infected persons.
${e://Field/data_storage}

${e://Field/costs_benefits}

${e://Field/actual_use}

Public health experts say that at least ${e://Field/percent_needed}% of US smartphone users need to use this app for it to be effective at limiting the spread of COVID-19. ${e://Field/expire_app}

---

ca_t Timing
First Click (1)
Last Click (2)
Page Submit (3)
Click Count (4)

End of Block: Conjoint Analysis

Start of Block: Conjoint analysis reading check and reflection

Display This Question:

If explainer_graphic != 1

JS

readagain_ng1
Click here to read about the app again
${e://Field/ca_nographic}

Display This Question:

If explainer_graphic = 1

JS

readagain_g1
Click here to read about the app again
${e://Field/ca_graphic}

---

recall_check From what you just read, please select all the **true statements** about the app below.

▢ The app will track your location data. (1)

▢ The app will send you the names of infected people you have been in close contact with. (2)

▢ All user data will be stored on a central server. (3)

▢ Apple and Google are building this app. (4)

▢ Public health experts say that at least 60% of smartphone users needs to use this app for it to be effective at limiting the spread of COVID-19. (5)

Page Break

Display This Question:

If explainer_graphic != 1

And thoughts_app = 1

JS

readagain_ng2
Click here to read about the app again
${e://Field/ca_nographic}

Display This Question:

If explainer_graphic = 1

And thoughts_app = 1

JS

readagain_g2
Click here to read about the app again
${e://Field/ca_graphic}

Display This Question:

If thoughts_app = 1

thoughts_app From what you just read, what are your thoughts on the app? What are the potential benefits of the app? What are the potential downsides of the app?
Please take 30 seconds to write down your reflections below.

____

____

____

____

____

Display This Question:

If thoughts_app = 1

X→

easy_understand How easy to understand was the description of the app?

- ◯ Very easy to understand (2)
- ◯ Somewhat easy to understand (1)
- ◯ Somewhat difficult to understand (-1)
- ◯ Very difficult too understand (-2)

Display This Question:

If easy_understand = -1

Or easy_understand = -2

thoughts_understand What did you find difficult to understand in the description of the app?

____

____

____

____

____

---

recall_check_t Timing
First Click (1)
Last Click (2)
Page Submit (3)
Click Count (4)

End of Block: Conjoint analysis reading check and reflection

Start of Block: Conjoint analysis main questions

Display This Question:

If explainer_graphic != 1

JS

readagain_ng3
Click here to read about the app again
${e://Field/ca_nographic}

Display This Question:

If explainer_graphic = 1

JS

readagain_g3
Click here to read about the app again
${e://Field/ca_graphic}

Display This Question:

If cellphone = 1

And cellphone_type != 5

download_likely ${e://Field/notification_download} On a 0 to 100 scale, how likely are you to **download this app and use it** while going outside your home?

Input a number below. 0 means extremely unlikely; 100 means extremely likely.

____

Display This Question:

If cellphone = 1

And cellphone_type != 5

report_likely On a 0 to 100 scale, How likely are you to **report to this app** if you tested positive for COVID-19?

Input a number below. 0 means extremely unlikely; 100 means extremely likely.

____

---

guess_perc By your best guess, what percentage of people in your town or city would use this app?

____

Page Break

Display This Question:

If explainer_graphic != 1

readagain_ng4
Click here to read about the app again
${e://Field/ca_nographic}

Display This Question:

If explainer_graphic = 1

readagain_g4
Click here to read about the app again
${e://Field/ca_graphic}

---

app_outcome **If enough people in the US population were to use this app, do you think it would...**

	Yes (1)	No (0)	Don't know (-88)
Help limit the spread of COVID-19 (1)	○	○	○
Improve the economy (2)	○	○	○
Make it safer for workers to return to work (3)	○	○	○
Make it safer for students to return to school (4)	○	○	○
Make it safer for me to visit friends and family (5)	○	○	○
Violate people's privacy (6)	○	○	○
Violate people's civil liberties (7)	○	○	○
Threaten US democracy (8)	○	○	○
Make tech companies too powerful (9)	○	○	○

confident_data How confident that your **personal data** will be protected if you download this app and use it?

- Very confident (3)
- Somewhat confident (2)
- Not very confident (1)
- Not confident at all (0)

End of Block: Conjoint analysis main questions

Start of Block: Conjoint analysis agreement block

Display This Question:

If explainer_graphic != 1

JS

readagain_ng5
Click here to read about the app again
${e://Field/ca_nographic}

Display This Question:

If explainer_graphic = 1

JS

readagain_g5
Click here to read about the app again
${e://Field/ca_graphic}

X→

gov_use_app Do you agree or disagree with the following statement?
${e://Field/government_level_q} should require everyone who has a smartphone to use this app.

- ◯ Strongly agree (2)
- ◯ Somewhat agree (1)
- ◯ Neither agree nor disagree (0)
- ◯ Somewhat disagree (-1)
- ◯ Strongly disagree (-2)

X→

auto_install_app Do you agree or disagree with the following statement?

Apple and Google should automatically install this app on users' iPhones or Android phones as part of a software update.

- Strongly agree (2)
- Somewhat agree (1)
- Neither agree nor disagree (0)
- Somewhat disagree (-1)
- Strongly disagree (-2)

---

empl_use_app Do you agree or disagree with the following statement?
Employers should require their employees with smartphones to use this app.

- Strongly agree (2)
- Somewhat agree (1)
- Neither agree nor disagree (0)
- Somewhat disagree (-1)
- Strongly disagree (-2)

---

rel_use_app Do you agree or disagree with the following statement?
Places of religious worship, like churches, synagogues, and mosques, should require everyone who has a smartphone to use this app if they want to worship there.

- Strongly agree (2)
- Somewhat agree (1)
- Neither agree nor disagree (0)
- Somewhat disagree (-1)
- Strongly disagree (-2)

---

obligation_report Do you agree or disagree with the following statement?
App users should have a choice in sharing their test outcomes with the app if they tested positive for COVID-19.

- Strongly agree (2)
- Somewhat agree (1)
- Neither agree nor disagree (0)
- Somewhat disagree (-1)
- Strongly disagree (-2)

---

ca_q_t Timing
First Click (1)
Last Click (2)
Page Submit (3)
Click Count (4)

End of Block: Conjoint analysis agreement block

Start of Block: Vaccine acceptance

Display This Question:

If vaccine = 0

vaccine_A As you may know, scientists around the world are racing to develop a vaccine for Covid-19 that would **protect people from being infected by the virus**.

When such a vaccine becomes available, do you plan to get vaccinated?

- Yes (1)
- No (-1)
- I'm not sure (0)

Display This Question:

If vaccine = 1

vaccine_B As you may know, scientists around the world are racing to develop a vaccine for Covid-19 that would **protect people who become infected by the virus from severe symptoms**, such as pneumonia, hospitalization, and death.

When such a vaccine becomes available, do you plan to get vaccinated?

- Yes (1)
- No (-1)
- I'm not sure (0)

Page Break

vaccine_manipulation The hypothetical Covid-19 vaccine you just read about would protect vaccinated individuals from...

- Becoming infected with Covid-19 (1)
- From experiencing severe symptoms from Covid-19 (4)
- I'm not sure (5)

End of Block: Vaccine acceptance

Start of Block: Demographics

X→

bornyear In what year were you born?

▼ 2003 (1) ... 1920 (84)

X→

gen What is your gender?

- Male (1)
- Female (2)
- Other (3)

---

race What is your race or ethnicity? You can select more than one.

- ▢ White (1)
- ▢ Black or African American (2)
- ▢ Hispanic or Latino (3)
- ▢ Native American or American Indian (4)
- ▢ Asian or Pacific Islander (5)
- ▢ Other (6) ____

---

demo_t_1 Timing
First Click (1)
Last Click (2)
Page Submit (3)
Click Count (4)

Page Break

state In which state do you currently reside?

▼ Alabama (1) ... I do not reside in the United States (53)

---

*

zipcode What is the zip code of your current residence?

____

---

householdsize Including yourself, how many people currently live in your household?

▼ 1 (1) ... 7 or more (7)

---

demo_t_2 Timing
First Click (1)
Last Click (2)
Page Submit (3)
Click Count (4)

Page Break

edu What is the highest level of school you have completed or the highest degree you have received?

- ○ Less than high school degree (1)
- ○ High school graduate (high school diploma or equivalent including GED) (2)
- ○ Some college but no degree (3)
- ○ Associate degree in college (2-year) (4)
- ○ Bachelor's degree in college (4-year) (5)
- ○ Master's degree (6)
- ○ Doctoral degree (7)
- ○ Professional degree (JD, MD) (8)

---

income What was your total household income last year?

▼ Less than $20,000 (1) ... More than $160,000 (9)

---

demo_t_3 Timing
First Click (1)
Last Click (2)
Page Submit (3)
Click Count (4)

Page Break

pid Generally speaking, do you usually think of yourself as a Republican, Democrat, Independent or something else?

- Republican (1)
- Democrat (2)
- Independent (3)
- Other party (4) ____

Page Break

Display This Question:

If pid = 2

strength_d Do you consider yourself a strong Democrat or not?

- Strong Democrat (1)
- Not strong Democrat (0)

Page Break

Display This Question:

If pid = 1

strength_r Do you consider yourself a strong Republican or not?

- Strong Republican (1)
- Not strong Republican (0)

Page Break

Display This Question:

If pid != 1

And pid != 2

lean As of today, do you lean more toward the Republican Party, the Democratic Party, or neither?

- Lean Republican (1)
- Lean Democrat (2)
- No preference (3)

Page Break

pol_ideology In general, would you describe your political views as...

- ○ Very conservative (1)
- ○ Conservative (2)
- ○ Moderate (3)
- ○ Liberal (4)
- ○ Very liberal (5)

Page Break

vote2016 Did you vote in the US presidential election in 2016?

- Yes (1)
- No (0)
- Prefer not to say (-77)

Display This Question:

If vote2016 = 1

votechoice2016 Which presidential candidate did you vote for in 2016?

- Donald Trump (1)
- Hillary Clinton (2)
- Other (3) ____
- Prefer not to say (-77)

vote2016_t Timing
First Click (1)
Last Click (2)
Page Submit (3)
Click Count (4)

Page Break

card_fraud In the past 12 months, have you had someone put fraudulent charges on your credit or debit card?

- Yes (1)
- No (0)
- Not sure (-88)
- Prefer not to say (-77)

Page Break

employment_status Which option best describes your current employment status?

- Employed full time (1)
- Employed part time (2)
- Unemployed looking for work (3)
- Unemployed not looking for work (4)
- Retired (5)
- Student (6)
- Disabled (7)
- Prefer not to say (-77)

employment_time Timing
First Click (1)
Last Click (2)
Page Submit (3)
Click Count (4)

Page Break

healthinsurance Do you currently have health insurance?

- Yes (1)
- No (0)
- Don't know (-88)
- Prefer not to say (-77)

Page Break

Display This Question:

If healthinsurance = 1

insurance_type What kind of health insurance do you have?

- ○ Private: employer-provided (1)
- ○ Private: self-bought (2)
- ○ Public: Medicaid (3)
- ○ Public: Medicare (4)
- ○ Other (5) ____
- ○ Don't know (-88)

Page Break

health_conditions Do you have any of the following health conditions?

	Yes (1)	No (0)	Prefer not to say (-77)
Diabetes (1)	○	○	○
High blood pressure (2)	○	○	○
Heart problems (3)	○	○	○
Asthma or other breathing problems (4)	○	○	○
Suppressed immune system (5)	○	○	○

Page Break

smoke Do you smoke cigarettes or vape?

- Neither (1)
- I smoke cigarettes only (2)
- I vape only (3)
- I smoke cigarettes and vape (4)
- Prefer not to say (-77)

Page Break

blm_movement From what you’ve read and heard, how do you feel about the Black Lives Matter movement?

- Strongly support (1)
- Somewhat support (4)
- Somewhat oppose (5)
- Strongly oppose (6)

---

racialequality Have you ever done any of the following in the past 2 months? Select all that apply.

- Contacted a public official to express your opinion on issues related to race or racial equality (1)
- Contributed money to a group or organization that focuses on race or racial equality (4)
- Attended a protest or rally that focused on issues related to race or racial equality (5)
- Had conversations with family or friends about issues related to race or racial equality (6)
- Posted or shared content on social networking sites related to race or racial equality (7)
- ⊗None of the above (8)

End of Block: Demographics

Start of Block: Final

Q118 We are coming to the end of the survey. Thank you very much for your time. Before you finish, we have two final questions.

---

num_covid_surveys How many surveys related to COVID-19 have you participated in, NOT including this survey? If you can't recall exactly, please give your best guess.

____

---

comments Did you experience any problems while taking the survey? Do you have any comments for the researchers?

____

____

____

____

____

End of Block: Final
